# Inactivation Kinetics of *Coxiella burnetii* During High-Temperature Short-Time Pasteurization of Milk

**DOI:** 10.3389/fmicb.2021.753871

**Published:** 2022-01-06

**Authors:** Marcel Wittwer, Philipp Hammer, Martin Runge, Peter Valentin-Weigand, Heinrich Neubauer, Klaus Henning, Katja Mertens-Scholz

**Affiliations:** ^1^Institute of Bacterial Infections and Zoonoses, Friedrich-Loeffler-Institut, Jena, Germany; ^2^Federal Research Institute of Nutrition and Food, Department of Microbiology and Biotechnology, Max Rubner-Institut, Kiel, Germany; ^3^Food and Veterinary Institute, Braunschweig/Hannover, Lower Saxony State Office for Consumer Protection and Food Safety, Hanover, Germany; ^4^Institute for Microbiology, University of Veterinary Medicine Hannover, Hanover, Germany

**Keywords:** *Coxiella burnetii*, milk, HTST pasteurization, *D*-/*z*-value, food safety

## Abstract

The Gram-negative, obligate intracellular bacterium *Coxiella burnetii* is the causative organism of the zoonosis Q fever and is known for its resistance toward various intra- and extracellular stressors. Infected ruminants such as cattle, sheep, and goats can shed the pathogen in their milk. Pasteurization of raw milk was introduced for the inactivation of *C. burnetii* and other milk-borne pathogens. Legal regulations for the pasteurization of milk are mostly based on recommendations of the Codex Alimentarius. As described there, *C. burnetii* is considered as the most heat-resistant non-spore-forming bacterial pathogen in milk and has to be reduced by at least 5 log_10_-steps during the pasteurization process. However, the corresponding inactivation data for *C. burnetii* originate from experiments performed more than 60 years ago. Recent scientific findings and the technological progress of modern pasteurization equipment indicate that *C. burnetii* is potentially more effectively inactivated during pasteurization than demanded in the Codex Alimentarius. In the present study, ultra-high heat-treated milk was inoculated with different *C. burnetii* field isolates and subsequently heat-treated in a pilot-plant pasteurizer. Kinetic inactivation data in terms of *D*- and *z*-values were determined and used for the calculation of heat-dependent log reduction. With regard to the mandatory 5 log_10_-step reduction of the pathogen, the efficacy of the established heat treatment regime was confirmed, and, in addition, a reduction of the pasteurization temperature seems feasible.

## Introduction

*Coxiella burnetii* is an obligate intracellular, Gram-negative bacterium and the causative agent of the zoonosis Q fever (Maurin and Raoult, 1999). It has a wide host range, including ruminants such as cattle, sheep, and goats. These animals are considered as the main source of infection for humans (Baca and Paretsky, 1983; Marrie and Raoult, 1997; Roest et al., 2011). During infection, *C. burnetii* replicates within a parasitophorous vacuole with phagolysosomal characteristics inside the host cell. Upon uptake, the bacteria differentiate from the spore-like cell form [small cell variant (SCV)] to the metabolically active and replicative cell form [large cell variant (LCV)] (McCaul and Williams, 1981; Coleman et al., 2004). The SCV is assumed to be more resistant to mechanical and osmotic stressors than the LCV (McCaul and Williams, 1981; Sandoz et al., 2016).

Infected animals can shed *C. burnetii via* their birth products (Bildfell et al., 2000; Arricau Bouvery et al., 2003), vaginal mucus (Berri et al., 2001; Bauer et al., 2020), feces (Guatteo et al., 2006), and milk (Schaal, 1980). The inhalation of contaminated aerosols is regarded as the main infection route (Welsh et al., 1958; Nusinovici et al., 2017). The infection risk *via* the oral route by consumption of *C. burnetii*-contaminated milk is considered to be much lower than inhalation but may lead to seroconversion (Marmion and Stoker, 1958; Benson et al., 1963; Fishbein and Raoult, 1992). However, a correlation between oral uptake and infection is still under discussion (Cerf and Condron, 2006; Miller et al., 2020).

Due to the zoonotic nature of the microorganism and its resistance against environmental stressors, including heat resistance, *C. burnetii* is considered one of the most persistent non-spore-forming bacterial pathogens in milk that can harm humans (McCaul and Williams, 1981; Codex Alimentarius, 2009; Roest et al., 2013). The currently applied pasteurization regimes for milk and fluid milk products are based on heat inactivation data of *C. burnetii* as determined by Enright et al. (1957). In this study, two different temperature–time combinations for heat inactivation of the pathogen in milk were used: high-temperature short-time treatment (HTST) at 72°C for 15 s and low-temperature long-time treatment at 63°C for 30 min. Both treatments resulted in a minimum 5 log_10_-step reduction of *C. burnetii* during pasteurization (Enright et al., 1957). The achieved 5 log_10_-step reduction of the pathogen was later adopted by the Codex Alimentarius and established as a performance criterion for both HTST and low-temperature long-time treatment of milk (Codex Alimentarius, 2009).

A recent reevaluation of the experiments of Enright et al. (1957) predicted a reduction of *C. burnetii* between 4.7 and 8 log_10_-steps (Cerf and Condron, 2006). This partly exceeds the requirements of the Codex Alimentarius. Considering the current pasteurization regimes, Hammer et al. (2015) demonstrated an 18 log_10_-step reduction of the highly heat-resistant bacterium *Mycobacterium bovis* (ssp. *bovis* and *caprae*). State-of-the-art industrial-scale pasteurizers are more efficient due to optimized plate heat exchangers and accelerated turbulent flow, compared with the older models used in 1957. Therefore, similar results could be expected for *C. burnetii* as shown for *M. bovis*. Assuming that a temperature reduction during pasteurization could be feasible, while maintaining a 5-log_10_ reduction of *C. burnetii*, the impact for the dairy industry (carbon dioxide footprint) and consumers (taste) could be perceptible.

The objective of this study was to evaluate the currently applied HTST-pasteurization process. Six *C. burnetii* isolates originating from cattle, goats, and sheep were used. The experimental setup was tested with the avirulent *C. burnetii* Nine Mile phase II RSA 439 (NMII) strain. To determine the inactivation kinetics for *C. burnetii*, a pilot-plant pasteurizer reflecting the heating technology currently applied in the dairy industry was used for identification of specific breakpoints (defined as the temperature where significant inactivation started, but a number of survivors would allow generation of kinetic inactivation data). Based on the result of their breakpoints, isolates with the highest heat resistance from each animal origin were selected for determination of *D*- and *z*-values (*D*-value: decimal reduction time; time required for a 1−log_10_ reduction of *C. burnetii* at a specific temperature; *z*-value: temperature increase necessary for a 1−log_10_ reduction of the *D*-value) by using a log-linear regression model. The corresponding data were used for the calculation of the heat-dependent log reduction.

## Materials and Methods

### Bacterial Isolates and Growth Conditions

*Coxiella burnetii* Nine Mile phase II RSA 439, kindly provided from L. Skultety (Slovak Academy of Science, Bratislava, Slovakia), and six German field isolates (strain collection Friedrich-Loeffler-Institut, Jena, Germany) were used in this study. Field isolates originated from cattle abortion or afterbirth material [Bru180 (18QC1770), M (18QC1771)], ovine abortion material [S1 (18QC1772), WDK1188 (18QC1773)], and caprine abortion or afterbirth material [WDK2932 (18QC1774), WDK299 (18QC1775)]. Except for isolate S1, the plasmid type and genotype of the field isolates were known. The investigated isolates carried the QpH1 plasmid and were grouped to the genotypes A1 (M, WDK2932, WDK299), A5 (WDK1188), and C2 (Bru180) (Frangoulidis et al., 2014). All field isolates and NMII were grown in buffalo green monkey cells or mouse fibroblasts (L-929) and subsequently propagated in acidified citrate cysteine medium (ACCM-2, Sunrise Science Products, San Diego, CA, United States) under biosafety level 3 conditions as described elsewhere (Coleman et al., 2004; Omsland et al., 2011). Briefly, buffalo green monkey or L-929 cells were inoculated with a multiplicity of infection of 50. Cell cultures were harvested when at least 80% of the cells showed *C. burnetii*-containing vacuoles and used for inoculation of ACCM-2. The axenic medium was inoculated with 6.8 × 10^2^ to 2.7 × 10^5^ GE/ml [genome equivalents, determined by quantitative real-time polymerase chain reaction (qPCR) as described later], and samples were taken at days 0, 3, 5, 7, and 10 for determination of growth curves. After 10 days, bacteria were harvested by centrifugation (10,000 × *g*, 15 min, 4°C) and stored in sucrose/glycerol buffer (270-mM sucrose, 10% glycerol) at −40°C.

### Quantification of *Coxiella burnetii* Using Quantitative Real-Time Polymerase Chain Reaction

For quantification, bacteria were lysed in 180 μl lysis buffer [250 mM Tris-hydrochloric acid, pH 7.5; 10 mM ethylenediaminetetraacetic acid; 4 g/l lysozyme (Brennan and Samuel, 2003)] and 20 μl proteinase K (22.2 mg/ml, Roche, Mannheim, Germany) for 1 h at 56°C and 300 rpm. DNA was isolated using the High Pure PCR Template Preparation Kit (Roche, Mannheim, Germany) according to the manufacturer’s instructions. Quantification was carried out by qPCR targeting the *icd* (isocitrate dehydrogenase) gene as described previously (Klee et al., 2006).

### Heat Treatment

Heat treatment was carried out under biosafety level 3 conditions, using a pilot-plant pasteurizer (Hammer et al., 2002). For each run, a total of 24 l commercially available ultra-high-temperature-treated (UHT) milk (3.5% milkfat, 6°C inoculation temperature) was inoculated with 1 × 10^10^ GE/L of *C. burnetii* isolates. Following the heating process, all milk samples (7–8 ml, 12°C sampling temperature) were taken separately using monovettes (Sarstedt, Nümbrecht, Germany), stored at 4°C for at least 30 min and subsequently transferred to −80°C.

Holding times and temperature profiles during the heating process differed depending on the investigation purpose and are summarized in Table 1. Breakpoint determination for the avirulent *C. burnetii* NMII strain, as well as for the six field isolates, was performed once (see Table 1). In contrast, determinations of the *D*-value of the most heat-resistant isolates (based on their breakpoints) from cattle, sheep, and goats were conducted three times, respectively (see Table 1). Non-heated, inoculated samples were used as a positive control for viability determinations.

**TABLE 1:** Time and temperature profiles during heat treatment.

Experiment	Strain/isolate	Heating temperature (°C)	Holding time (s)
Breakpoint determination^1^	NMII^3^	75, 72.5, 70, 67.5, 65, 62.5, 60	20
	NMII^4^	75, 71.5, 68, 64.5, 61, 57.5, 54	20
	M, Bru180, WDK299, WDK2932, WDK1188, S1	75, 71.5, 68, 64.5, 61, 57.5, 54	20
*D*-value determination^2^	M, WDK299, WDK1188	65, 62.5, 60	15, 20, 25

*^1^Performed once for each isolate.*

*^2^Performed three times for each isolate, three holding times at each heating temperature.*

*^3^NMII grown in ACCM-2.*

*^4^NMII grown in cell culture.*

### Viability Testing of *Coxiella burnetii* After Heat Treatment

Heated samples were analyzed for viable *C. burnetii* cells using determination of colony-forming units (CFUs) and inoculation of embryonated chicken eggs (Samuel and Hendrix, 2009; Omsland et al., 2011).

Colony-forming units were determined as described by Omsland et al. (2011). Briefly, agarose/ACCM-2 plates were poured by mixing 7,5 ml double-concentrated ACCM-2 with 7,5 ml pre-warmed 2% agarose. Aliquots of heated 1% agarose were kept at 58°C and 300 rpm. Samples and controls (positive = non-heated, inoculated sample; negative = non-inoculated UHT milk with 3.5% milk fat) were serially diluted in 2 × ACCM-2. The diluted samples were mixed with an equal amount of 1% agarose, and two 100 μl drops of each sample mixture were immediately applicated onto agarose-ACCM-2 plates. Plates were incubated at 37°C, 5% carbon dioxide, and 2.5% oxygen (oxygen was displayed by nitrogen gas) for 7 days, resulting in flat agarose drops with distinct and clearly separated colonies. For CFU/ml calculation, the double determinated colonies of at least two different dilutions were counted (IX70, Olympus, Tokyo, Japan; 40-fold magnification).

Clean eggs (VALO BioMedia GmbH, Osterholz-Scharmbeck, Germany) were used for inoculation of heated *C. burnetii* samples, according to Samuel and Hendrix (2009). Non-inoculated UHT milk (3.5% milkfat) was used as negative control and the non-heated sample as positive control. Briefly, the inoculation site of the egg was disinfected with 70% ethanol and pierced with a sterile lancet needle. Milk samples (100 μl) were injected into the yolk sac, and subsequently, the egg was sealed with a solvent-free instant adhesive (multipurpose glue, UHU, Bühl, Germany). Eggs were incubated at 38°C and 65% humidity while permanent slowly rotating (12 revolutions per day). The embryos were checked every 2 days with a candling lamp and harvested 11 days post-inoculation or in case of premature death (no embryo growth, dark discoloration, and fewer blood vessels). All eggs were placed overnight at 4°C before harvest. The yolk sac membranes were separated, rinsed with phosphate-buffered saline (pH 7.2, Merck, Darmstadt, Germany), and homogenized at 6,000 rpm for 2 min (DT-50-M/Ultra Turrax Tube Drive Control, IKA, Staufen, Germany). Aliquots of 100 μl were taken for DNA isolation and quantification by qPCR.

### Statistics Analysis and Calculations

The normal distribution of the investigated samples was estimated using the Shapiro–Wilk test.

For significance predictions, the Mann–Whitney *U* test (no normal distribution) or the two-tailed, unpaired two-sample *t*-test (samples normally distributed) was used. For the latter, samples were first analyzed with an *F*-test to control the equality of variances of tested pairs.

The surviving fraction after heat treatment was (S/S_0_) determined as the quotient of colony counts (CFU/ml) of surviving bacteria at a specific temperature and untreated bacteria.

For calculation of the *D*- and *z*-values, the results of the CFU assay were used for the log-linear regression model, according to Hammer et al. (2015). A first-order kinetic referring to Bigelow (1921) is assumed as the basic principle for the inactivation model. The log_10_ of the heated samples with visible colonies (count of survived bacteria; N_*t*_), divided by the positive control (initial count; N_0_), was calculated (referred to as “y” in the formula). The results were plotted against the holding time (referred to as “x”), and a log-linear regression curve was calculated. With the slope of the regression curve (referred to as “m”) as divisor and 1 as a dividend, the *D*-values were determined.

$$m=\frac{\sum_{i=1}^{\text{n}}\left(x_{i}-\bar{x}\right)\,\left(y_{i}-\bar{y}\right)}{\sum_{i=1}^{\text{n}}{\left(x_{i}-\bar{x}\right)}^{2}}$$

$$\bar{\text{x}}=\frac{1}{n}\,\sum_{i=1}^{n}x_{i}$$

$$\text{D-value}=\frac{1}{\text{m}}$$

In addition, the coefficient of determination (*R*^2^) for each regression curve was used to verify the fit of the model. The *z*-values were determined by plotting the log_10_-transformed *D*-values against heating temperature following calculations as described earlier.

Log increase during growth was calculated as the absolute value of the log_10_ of the starting inoculum (GE/ml; N_0_), divided by the bacterial amount after 10 days of growth (GE/ml; N_*t*_).

Statistical outliers were determined using Dixon’s Q test and Grubbs’s test.

For all calculations, Excel 2016 (Microsoft Corporation, Redmond, WA, United States) and SAS EG (SAS Institute, Cary, NC, United States) were used.

## Results

### Preliminary Studies With *Coxiella burnetii* Nine Mile Phase II

The experimental setup for heat treatment of *C. burnetii* field isolates in the pilot-plant pasteurizer was established in preliminary experiments using *C. burnetii* NMII (Wittwer, 2020). The optimal temperature profile for breakpoint determination of the field isolates and differences in heat sensitivity of ACCM-2 and cell culture grown NMII was determined. In addition, the occurrence of the spore-like cell form (SCV) of ACCM-2 grown NMII was investigated using transmission electron microscopy (TEM).

According to the CFU assay, the breakpoints of ACCM-2 and cell culture-grown NMII were identical with 65 and 64.5°C, respectively (Supplementary Figures I, II). The difference of 0.5°C was caused by the applied heating profiles due to experimental reasons. The data showed that growth in ACCM-2 had no negative effect on heat resistance compared with cell culture-propagated NMII.

After 10 days of growth in ACCM-2, the presence of the SCV cell form was detected, according to TEM (Supplementary Figure IIIA). The dimensions of the corresponding SCVs were compared with the spore-like cell forms of an SCV-enriched NMII culture after 8 weeks of growth in ACCM-2 (Supplementary Figure IIIB). Altogether, the length and width of SCVs after 10 days ($\bar{x}$ = 0.6/0.36 μm) and 8 weeks of growth ($\bar{x}$ = 0.64/0.31 μm) were comparable. However, the appearance of intermediate forms made it difficult to clearly identify the percentage of SCVs after 10 days of growth in the axenic medium.

### Growth of *Coxiella burnetii* Field Isolates in Axenic Medium

**FIGURE 1:**
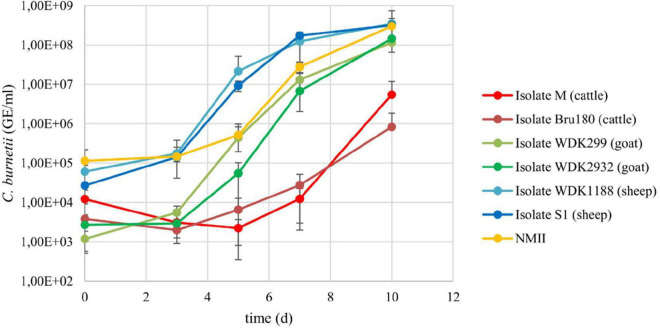
Growth curves of the six *Coxiella* field isolates and *C. burnetii* NMII in ACCM-2. All isolates were inoculated with 6.8 × 10^2^ to 2.7 × 10^5^ GE/ml and incubated for 10 days. Aliquots were taken regularly and bacteria quantified using qPCR. Results represent average of at least three independent experiments and respective standard deviations.

To exclude any differences regarding the ability to grow in axenic medium, the growth of the six *C. burnetii* field isolates and NMII was monitored over 10 days in ACCM-2 (Figure 1).

Isolates from the same animal species replicated at a similar rate as indicated by comparison of the log increase of the cattle (M = 2.66; Bru180 = 2.34), goat (WDK299 = 4.99; WDK2932 = 4.73), and sheep isolates (WDK1188 = 3.76; S1 = 4.08). The log increase of NMII was 3.42. The values of the cattle isolates were not log-normal distributed. Despite the longer lag-phase of the cattle isolates, statistical analysis revealed no significant difference between all *C. burnetii* isolates in their growth behavior in ACCM-2 (Supplementary Table I). The yield of the cattle isolate M was enhanced for the *D*-value determination by increasing the inoculation dose to 4 × 10^5^ GE/ml (referred to as “M2”). The corresponding growth curve was log-normal distributed (Supplementary Figure IV), and no significant difference compared with the other two isolates used in the main experiments was detected (data not shown).

In addition, the growth of the field isolates in the axenic medium and cell culture was compared. Referring to this, a double to 13-fold higher daily log increase was detected for ACCM-2 (Supplementary Table II).

Taken together, these data show that the growth behavior of the tested strains was comparable. The SCV content of the virulent isolates was not determined because their similar growth behavior, compared with the NMII strain, implied a similar SCV content.

### Breakpoint Determination of the Field Isolates

**FIGURE 2:**
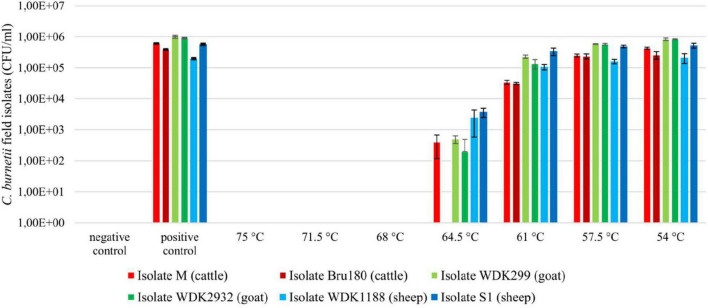
Viability testing of *Coxiella* field isolates using CFU assay, after heat treatment between 54 and 75°C. Inoculated UHT milk (3.5% milkfat; 1 × 10^7^ GE/ml) was heat-treated at a holding time of 20 s in a pilot-plant pasteurizer. Non-heated, inoculated samples served as positive control and non-inoculated UHT milk as negative control. Results represent calculated average and standard deviation originating from four values, respectively. Displayed pasteurization temperatures were summarized to their intended values, without the individual temperature variations of approximately ± 0.3°C.

After heat treatment of the field isolates, no viable bacteria were detectable at temperatures between 68 and 75°C, according to the CFU assay (Figure 2). Surviving bacteria were observed at 64.5°C. The only exception was the cattle field isolate Bru180, which was still inactivated under these conditions. Below 64.5°C, all isolates showed a similar survival pattern. Compared with the untreated positive control, there was partial heat inactivation at 61°C, but no inactivation was observed at 57.5°C.

The results of the viability testing of the isolates in embryonated chicken eggs are displayed for isolate M (Supplementary Figure V). Samples that depict significantly higher *C. burnetii* genome equivalents than their inoculation dose (approximately 1 × 10^6^ GE/egg) or rather the heat-inactivated bacteria suspension at 75°C were counted as viable. The results of the chicken egg experiments confirmed mostly the results of the CFU assay (remaining data not shown). Nevertheless, verification in embryonated chicken eggs was less accurate. This method was time-consuming, more error-prone and less precise than the CFU assay, and not used for *D*-value determination and subsequent *z*-value calculation of the field strains.

The calculation of the surviving fraction showed a similarity between the *C. burnetii* cattle and goat isolates (Supplementary Figure VI). The number of survivors of both sheep isolates was higher at tested temperatures. However, statistical analysis showed no significant difference between the surviving fractions of the field isolates (data not shown).

In summary, heat inactivation of the tested field isolates was comparable, except for the cattle isolate Bru180 at 64.5°C.

### Determination and Calculation of *D*- and *z*-Values

For *D*-value determination and *z*-value calculation, the most heat-resistant isolate from each animal species was selected. However, only the two cattle isolates showed a respective difference regarding their breakpoints (Figure 2). According to this, isolate M was chosen for the following experiments. Due to the similar growth capacities and the surviving fraction of the goat and sheep isolates, WDK299 and WDK1188 were selected randomly. With every isolate, three heating runs were performed, respectively (referred to as experiments A, B, and C). The corresponding results of the viability testing using CFU assay are shown in Supplementary Figures VII–IX for all three isolates.

Based on these results, three *D*-values were determined for each heating run and the respective *z*-value calculated. For a better understanding of the calculation process, relevant data for *D*- and *z*-value determination are exemplified for all tested isolates (Supplementary Figures X–XVIII and Supplementary Tables III–XI). The determination coefficients of the log-transformed survivors compared with the holding time showed a strong fit with the assumed log-linear first-order inactivation model (21 times *R*^2^ > 0.95; four times *R*^2^ > 0.9; one time *R*^2^ > 0.85). The *D*- and *z*-values for all three isolates are summarized in Table 2.

**TABLE 2:** *D*- and *z*-values of *Coxiella* isolates M, WDK299, and WDK1188.

*C. burnetii* isolate	Experiment	*D*-value (s)			*z*-Value (°C)
		65°C	62.5°C	60°C	
M (cattle)	A	7	21	42	6.4
	B	7.6	14.3	24.4	9.9
	C	7.6	15.5	/^1^	8.1
WDK299 (goat)	A	5.9	12.3	25	8
	B	5.5	15	45.9	5.4
	C^2^	16.6	78.7	169.5	4.9
WDK1188 (sheep)	A	6.9	19	51.6	5.7
	B	6.3	11.6	21.6	9.3
	C	5.1	10.6	21.3	8

*^1^No sample available.*

*^2^With a certainty of 99% (α = 0.01), it could be shown that experiment C with isolate WDK299 is a statistical outlier, according to Dixon’s Q test and Grubbs’s test.*

### Calculation of the Heat-Dependent Log Reduction

The *D*- and *z*-values of the isolates M, WDK299, and WDK1188 were used for the calculation of their corresponding heat-dependent log reduction. The results of isolate M in experiment B will be used as an example. For the calculation, a holding time of 15 s was used for better comparability with the HTST demands of the Codex Alimentarius.

A temperature enhancement of 9.9°C (according to the determined *z*-value of isolate M in experiment B) will reduce the corresponding *D*_62_._5_-value (14.3 s) to 1.43 s at a temperature of 72.4°C (62.5 + 9.9°C). Using a holding time of 15 s and dividing it by 1.43 s results in a predicted reduction of approximately 10.5 log_10_-steps at 72.4°C. This outperforms the 5 log_10_-step reduction at 72°C and 15-s holding time demanded by the Codex Alimentarius.

**FIGURE 3:**
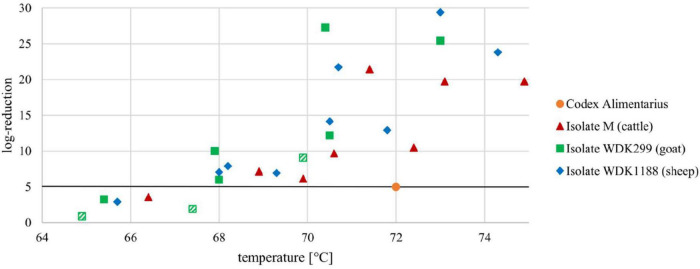
Heat-dependent log reduction of three *Coxiella* isolates M, WDK299, and WDK1188 during a holding time of 15 s. Black line represents mandatory 5 log_10_-step reduction of *C. burnetii*, as demanded in Codex Alimentarius. Orange dot illustrates HTST demands at 72°C. Statistical outliers of isolate WDK299 are depicted as hatched squares.

Based on the results of their *D*- and *z*-values, the heat-dependent log reduction of all measured isolates was calculated and is shown in Figure 3. Values above the mandatory 5 log_10_-step reduction indicate a successful pasteurization process.

## Discussion

The objective of the present study was to reevaluate the results for heat inactivation of *C. burnetii* established by Enright et al. (1957) as a “worst-case” scenario with respect to improved heating technology and detection methods for survivors. Before heat treatment of the *C. burnetii* field isolates in the pilot-plant pasteurizer, the experimental setup was established in preliminary experiments with *C. burnetii* NMII grown in cell culture and axenic medium. This revealed no major difference in the determined breakpoints between both ACCM-2 and cell culture-derived bacteria according to the CFU assay (Supplementary Figures I, II).

For the heating experiments, a large amount of *Coxiella* cells was required (2.4 × 10^11^ GE per run). All of the six investigated *C. burnetii* isolates were able to grow in an axenic medium, although propagation of the pathogen in ACCM-2 is not always certain (Kersh et al., 2016). Growth experiments of the *C. burnetii* field isolates performed in parallel exhibited even a double to 13-fold higher daily log increase during growth in ACCM-2 than compared with cell culture (Supplementary Table II). Considering the absence of significant growth differences of the *C. burnetii* field isolates in ACCM-2 (Figure 1 and Supplementary Table I), cultivation in the axenic medium was preferred to cell culture to achieve the vast amount of necessary *Coxiella* cells.

However, it is known that multiple passages in the axenic medium negatively affect the virulence of the pathogen (Kersh et al., 2011; Beare et al., 2018). To avoid this, established *C. burnetii* cell cultures of the field isolates served as basic raw material, followed by single passages in ACCM-2. The mentioned virulence-loss of the pathogen, which is associated with lipopolysaccharide truncation, does not occur during the first two (Beare et al., 2018) or rather eight passages (Kersh et al., 2011) in the axenic medium. These indicate that possibly appearing morphological changes that may affect the heat-dependent inactivation kinetics of the pathogen should be negligible during single passaging in ACCM-2.

In the present study, the commonly used incubation period for the cultivation of *C. burnetii* in the axenic medium was slightly increased. After 7 days of incubation in ACCM-2, the pathogen resides in the stationary growth phase (Omsland et al., 2011). Despite this, *C. burnetii* can maintain its viability in ACCM-2 for at least 21 days while increasing the portion of the spore-like form (Sandoz et al., 2014). Furthermore, a higher yield of the tested *C. burnetii* isolates was detected after 10 days of growth in ACCM-2, compared with 7 days (Figure 1). Taking all this information together, an incubation period of 10 days was chosen for the axenic propagation of the *Coxiella* isolates.

It is assumed that the SCV cell form is more heat resistant than the LCV. However, data supporting a higher heat resistance of the spore-like cell form are missing because corresponding studies were performed with mixed *C. burnetii* cell populations (Sandoz et al., 2016). Nevertheless, the SCV is more resistant to osmotic and mechanical stressors (McCaul and Williams, 1981; Sandoz et al., 2016), and for meaningful statements regarding the heat resistance of *C. burnetii* during pasteurization, the presence of the spore-like cell form in the heated samples was preferred. However, SCV determinations of the field strains were complicated due to their biosafety risk classifications. To that effect, the occurrence of SCVs of ACCM-2-grown NMII was investigated using TEM. The experiment confirmed the existence of SCVs after 10 days of growth in ACCM-2 (Supplementary Figure IIIA). Due to their similar growth phases (Figure 1 and Supplementary Figure IV) and in consideration of the corresponding statistics (Supplementary Table I), a comparable SCV content of the virulent field isolates and the NMII strain was assumed.

The determination of CFUs was more accurate and less time-consuming than quantification of the pathogen using embryonated chicken eggs. In scientific efficiency and animal welfare aspects (replace, reduce and refine principle), detection of *C. burnetii* in embryonated chicken eggs seems to be outdated and should be replaced by contemporary methods. However, a definite recommendation must be based on the results of appropriate designed future studies.

A point of criticism regarding the study of Enright et al. (1957) is the undefined origin of the used *C. burnetii* material and the lack of information on whether a single strain or a mixture was used during the heating experiments (Cerf and Condron, 2006). In contrast, isolates used in this study were characterized in their origin, growth, and MLVA genotype (Frangoulidis et al., 2014). Because of the importance of the animal host for the dairy industry, two isolates from cattle, goats, and sheep were chosen (Faye and Konuspayeva, 2012). Based on the determined breakpoints, the most heat-resistant isolate from each host species was selected for the next step of experiments; however, only the two cattle isolates showed a respective difference (Figure 2; 64.5°C: isolate M and Bru180).

In contrast to Enright et al. (1957), artificially inoculated UHT milk was used for all heating experiments. This guarantees a homogeneous matrix, and a bias due to contamination with competing flora is minimized. The prevalence of *C. burnetii* in ruminants is high, and shedding *via* the milk is common (Schaal, 1980; Baca and Paretsky, 1983; Marrie and Raoult, 1997; Roest et al., 2011). The here used UHT milk was not tested *via* PCR in advance because the CFU assay for verification of survivors detects viable *Coxiella* only. Milk producers claim commercial sterility with only 1 of 10,000 UHT milk packages (1 l) may be contaminated. Testing non-inoculated UHT milk as negative controls *via* CFU assay and inoculation of embryonated chicken eggs resulted in negative PCR results and no growth. This implies that UHT milk does not contain viable *C. burnetii*, and possible contamination with *C. burnetii*-specific DNA is very low and negligible.

A criticism of the here presented study might be the storage of heat-treated samples in an ultra-low freezer before analyses. It cannot be excluded that heat stress in advance of freezing may negatively impact the viability of *C. burnetii*. To avoid this, the heat-treated samples wrapped in cellulose tissue were cooled in a refrigerator to approximately 4°C for at least 30 min and transferred into a deep-freezer to achieve a slow freezing process. Due to logistics, it was not possible to transport the samples within 24 to 48 h. Therefore, freezing was the most suitable way to stably maintain the samples till processing.

The laboratory-generated results of Enright et al. (1957) were subsequently confirmed by the use of a commercial pasteurization plant. Here described heating experiments were performed in a pilot-plant pasteurizer mimicking the state-of-the-art equipment used in industrial practice (Peng et al., 2013; Hammer et al., 2015). The principle of the plate heat exchange and the temperature–time regimes are the same as applied in 1957. However, technical advancements during the last 60 years (more powerful pumps, optimally engineered heat exchangers, and higher turbulence) enable a faster and more effective heat transfer, resulting in an enhanced pathogen inactivation.

The study of Enright et al. (1957) was the first of its kind using regression analysis to describe the inactivation of *C. burnetii*, but the linearity of the survival curve was not investigated, resulting in an uncertain value for log reduction (Cerf and Condron, 2006). In line with Hammer et al. (2015), a regression model was used for *D*- and *z*-value calculation, which assumed a linear correlation during heat-dependent inactivation of *C. burnetii*. For better evaluation of the generated data regarding the predicted log-linearity, the coefficients of determination were calculated (Supplementary Figures X–XVIII). Of the 26 corresponding datasets, which were subsequently used for *D*-value calculation, 21 showed a strong fit with the assumed log-linear first-order inactivation model (*R*^2^ > 0.95). The fit of the remaining five datasets was still good in statistical criteria (four times *R*^2^ > 0.9; one time *R*^2^ > 0.85). The strong fit of the determination coefficients is in agreement with a study from Wagner et al. (2018) regarding the heat inactivation of the bacteriophage *Lactococcus lactis* conducted in the same pilot-plant pasteurizer. Taken together, the regression model and the here obtained data seemed to be suitable for an appropriate explanation of the heat inactivation of *C. burnetii*. Extrapolation that exceeds the obtained data can only deliver an estimate for proposed inactivation. However, in the case of higher temperatures, a stronger inactivation is to be expected, and even if linearity is not continuing, inactivation will be underestimated. This will add a margin of safety to the proposed log reductions.

After heat treatment of the *C. burnetii* field isolates in the pilot-plant pasteurizer, their corresponding *D*-values were determined. The *D*-values of all isolates showed comparable results within the tested conditions, with the exception of the goat isolate WDK299 in experiment C (Table 2). Nonetheless, Dixon’s Q test and Grubbs’s test revealed with a percentage of 99% (α = 0.01) that WDK299 experiment C is a statistical outlier. To that effect, WDK299 C will be excluded as an experimental mistake, although the high *D*-values of this isolate were put into perspective through its corresponding low *z*-value (Figure 3).

The heat-dependent log reduction during a holding time of 15 s was depicted for all selected isolates (Figure 3). As seen there, the mandatory 5 log_10_-step reduction of the pathogen for HTST treatment (72°C, 15 s holding time) was never underrun. Fortunately, the data indicate a 5 log_10_-step reduction even at a temperature of 68°C for all tested isolates, which is in line with our research hypothesis.

Comparison of the generated data with those in the study of Hammer et al. (2015) indicates a higher inactivation of *M. bovis* and *Mycobacterium caprae* (>18 log_10_-steps) during HTST treatment of milk than for *C. burnetii* (>10 log_10_-steps). This comparatively higher heat resistance is in agreement with the demand, that inactivation of *C. burnetii* should be the internationally accepted performance criterion for milk pasteurization (Codex Alimentarius, 2009). With regard to the precautionary principle, the presence of the spore-like cell form in the investigated samples is assumed, as already described. It can be suspected that the heat resistance of *C. burnetii* is influenced by the spore-like cell form of the bacterium, but this assumption must be clarified in further pasteurization studies.

## Conclusion

In summary, it was shown that the current HTST pasteurization regimes for the reduction of *C. burnetii* in milk and fluid milk products are valid. Additionally, *C. burnetii* is more intensively inactivated during HTST pasteurization of milk than required by the Codex Alimentarius. A temperature reduction seems to be feasible. According to the presented results, the performance criterion of 5 log_10_-step reductions for HTST treatment, as demanded by the Codex Alimentarius, can be achieved at temperatures above 68°C. However, the log reduction of the goat isolate WDK299 at 68°C and for the cattle isolate M at 70°C was barely above 5 log_10_-steps (Figure 3). In addition, we could show that the breakpoints and, accordingly, the heat resistance of *C. burnetii* isolates can differ (Figure 2). To that effect, the existence of more heat-resistant *Coxiella* isolates cannot be excluded. If reduction of temperature is considered, the practice needs to be evaluated by the respective industries. Less intense heat treatment could result in financial benefits for the dairy industry, but gains in reduced energy costs need to be calculated for each plant individually. Mainly, differences in technological equipment regarding the general capacity and performance of heat exchangers for heating and heat recovery must be included. The sensory difference in less heated milk should be detectable by a trained panel. Besides pathogenic microorganisms, temperature reduction would also be of impact on spoilage microorganisms. Studies on shelf life regarding the latter would be necessary for addition.

## Data Availability Statement

The original contributions presented in the study are included in the article/Supplementary Material, further inquiries can be directed to the corresponding author.

## Ethics Statement

Ethical review and approval was not required for the animal study because use of embryonated hen eggs without hatching is not an animal experiment according to the German Protection of Animal Act (Tierschutzgesetz, §14 Tierschutz-Versuchstierverordnung).

## Author Contributions

KH, PH, and KM-S designed the study. KH and PH arranged the funding. MW and PH performed the experiments and analyzed the data. MW and KM-S wrote the manuscript. MR and PV-W supervised the study. All authors contributed to the manuscript and approved the submitted publication.

## Conflict of Interest

The authors declare that the research was conducted in the absence of any commercial or financial relationships that could be construed as a potential conflict of interest.

## Publisher’s Note

All claims expressed in this article are solely those of the authors and do not necessarily represent those of their affiliated organizations, or those of the publisher, the editors and the reviewers. Any product that may be evaluated in this article, or claim that may be made by its manufacturer, is not guaranteed or endorsed by the publisher.
