# Supplementary material for: Inactivation Kinetics of Coxiella burnetii During High-Temperature Short-Time Pasteurization of Milk
Source: Front Microbiol. 2022 Jan 6;12:753871. doi: 10.3389/fmicb.2021.753871 (PMC8770862; doi:10.3389/fmicb.2021.753871)
Supplement: Supplementary file 1 [file Data_Sheet_1.docx]

**Supplement:**

Figures:


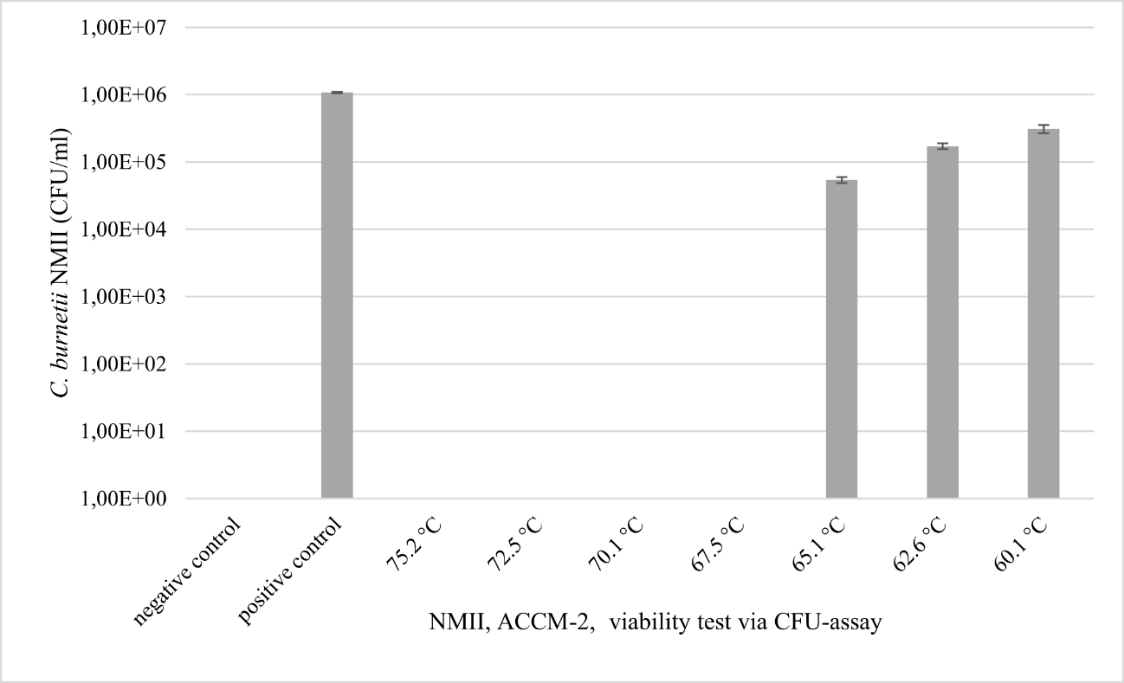


**Fig. I: Viability testing of an ACCM-2 grown, 10 days old *C. burnetii* NMII culture after heat treatment between 60 – 75 °C using CFU assay.** Inoculated UHT milk (3.5% milk fat; 1 x 10^7^ GE/ml) was heat treated at a holding time of 20 s in a pilot plant pasteurizer. A non-heated, inoculated sample was used as positive control and non-inoculated UHT milk as negative control. Results represent the calculated average and standard deviation from four values, respectively.


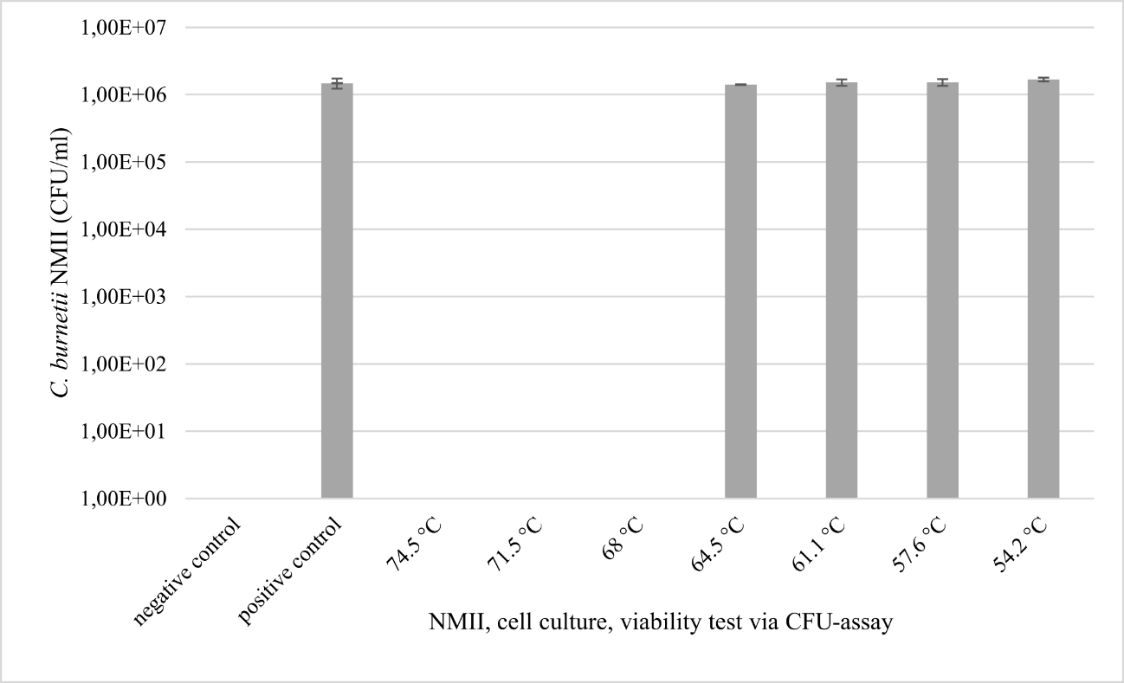


**Fig. II: Viability testing of cell culture grown *C. burnetii* NMII after heat treatment between 54 – 75 °C using CFU assay.** The cell culture was harvested when at least 80% of the cells showed *C. burnetii*-containing vacuoles (CCV). Inoculated UHT milk (3.5% milk fat; 1 x 10^7^ GE/ml) was heat treated at a holding time of 20 s in a pilot plant pasteurizer. A non-heated, inoculated sample was used as positive control and non-inoculated UHT milk as negative control. Results represent the calculated average and standard deviation from four values, respectively.


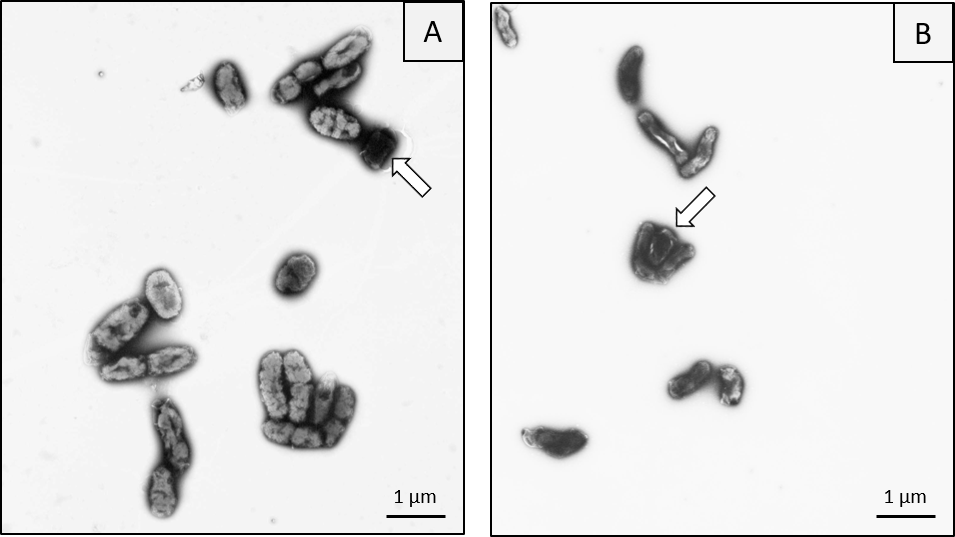


**Fig. III: Determination of the NMII SCV cell form after 10 days (A) and 8 weeks (B) of growth in ACCM-2.** The SCV cell form is highlighted with white arrows. The length and width of the SCV´s after 10 days (x̅ = 0.6/0.36 µm) and 8 weeks of growth (x̅ = 0.64/0.31 µm) were determined using ImageJ. Altogether, at least five different TEM images of each cultivation method were analyzed.


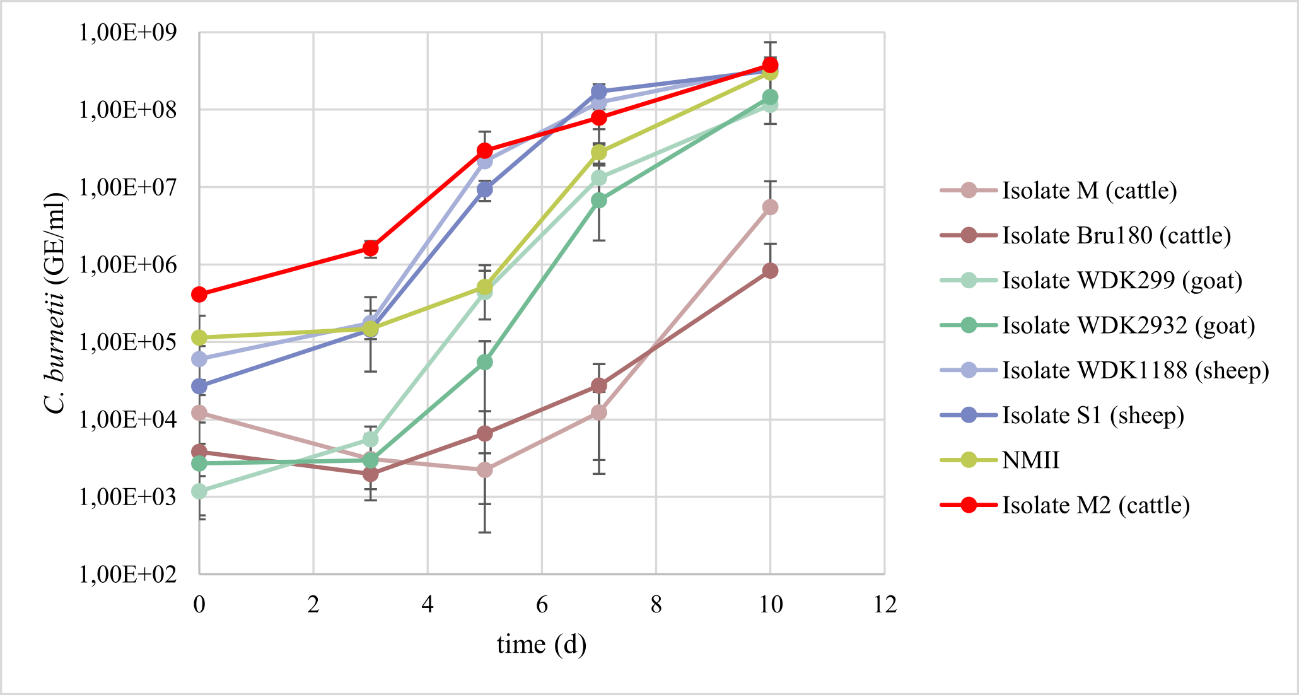


**Fig. IV: Growth curve of the cattle isolate M2 in comparison to the remaining field isolates as well as the NMII strain.** Isolate M2 was inoculated with 4 x 10^5^ GE/ml and incubated for 10 days. Aliquots were taken regularly and bacteria quantified using qPCR. Results represent the average of two independent experiments and respective standard deviations.


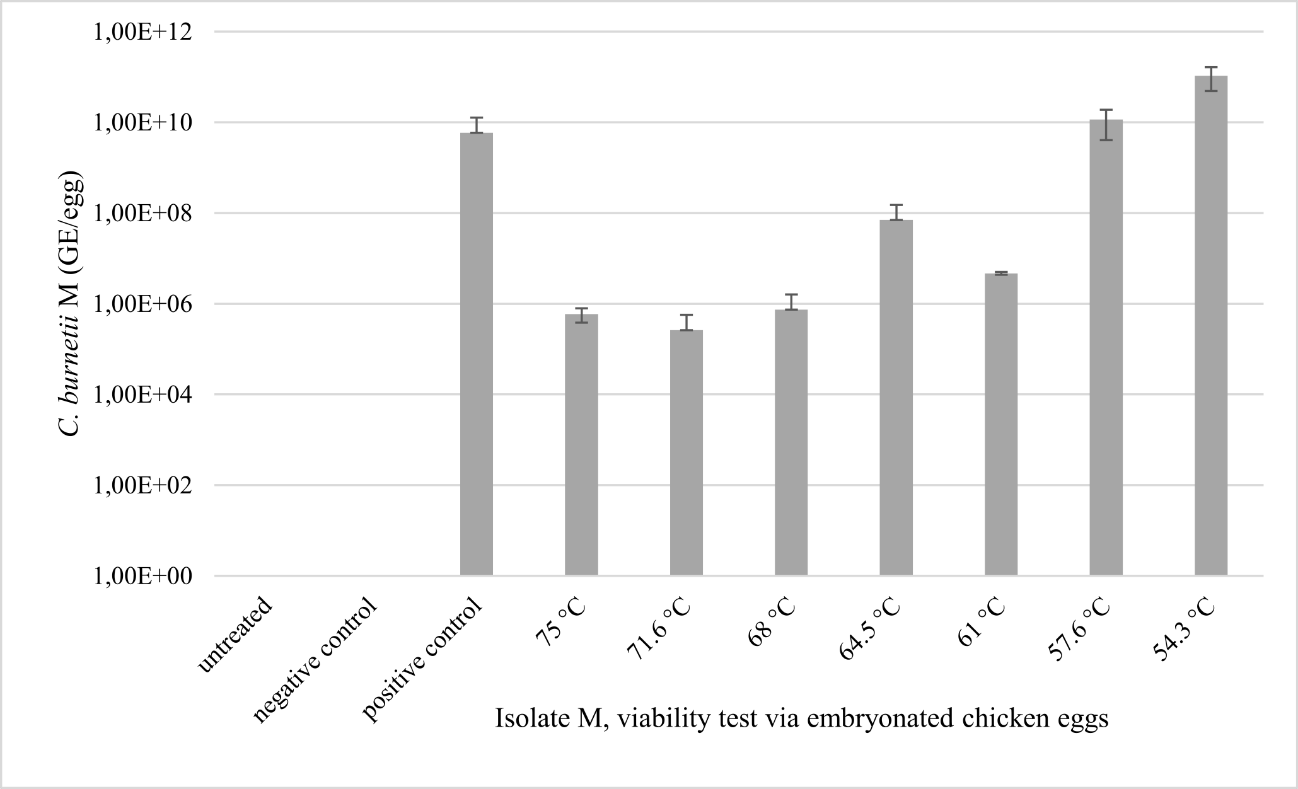


**Fig. V: Viability testing of *C. burnetii* isolate M in embryonated chicken eggs, after heat treatment between 54 – 75 °C.** Inoculated UHT milk (3.5% milk fat; 1 x 10^7^ GE/ml of isolate M) was heat treated at a holding time of 20 s in a pilot plant pasteurizer. Non-heated, inoculated samples served as positive control and non-inoculated UHT milk as negative control. Untreated eggs were used as second negative control. Results represent the calculated average and standard deviation originating from four to six Ct-values, respectively.


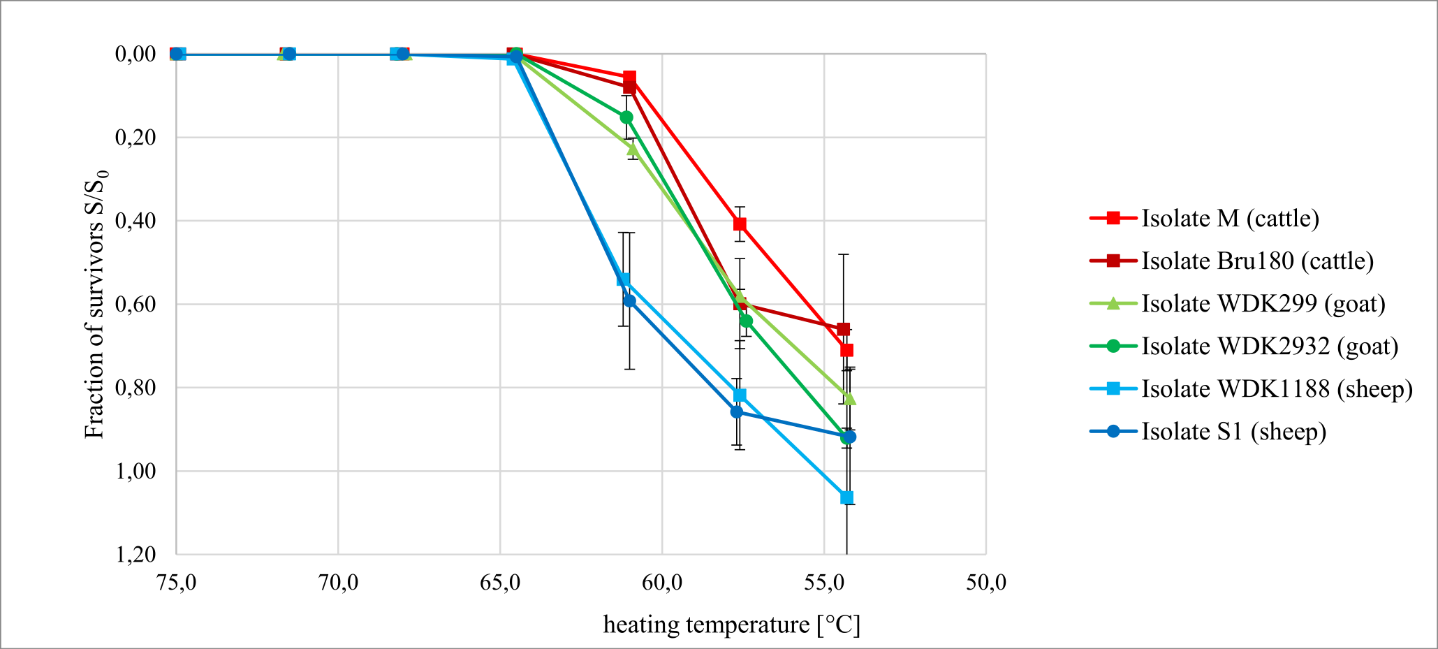


**Fig. VI: Survival of *C. burnetii* field isolates after heat treatment between 54 – 75 °C.** Presented results were calculated from the CFU assay data of the breakpoint determination (see Fig. 2) and represent the mean of four individual values and respective standard deviations.


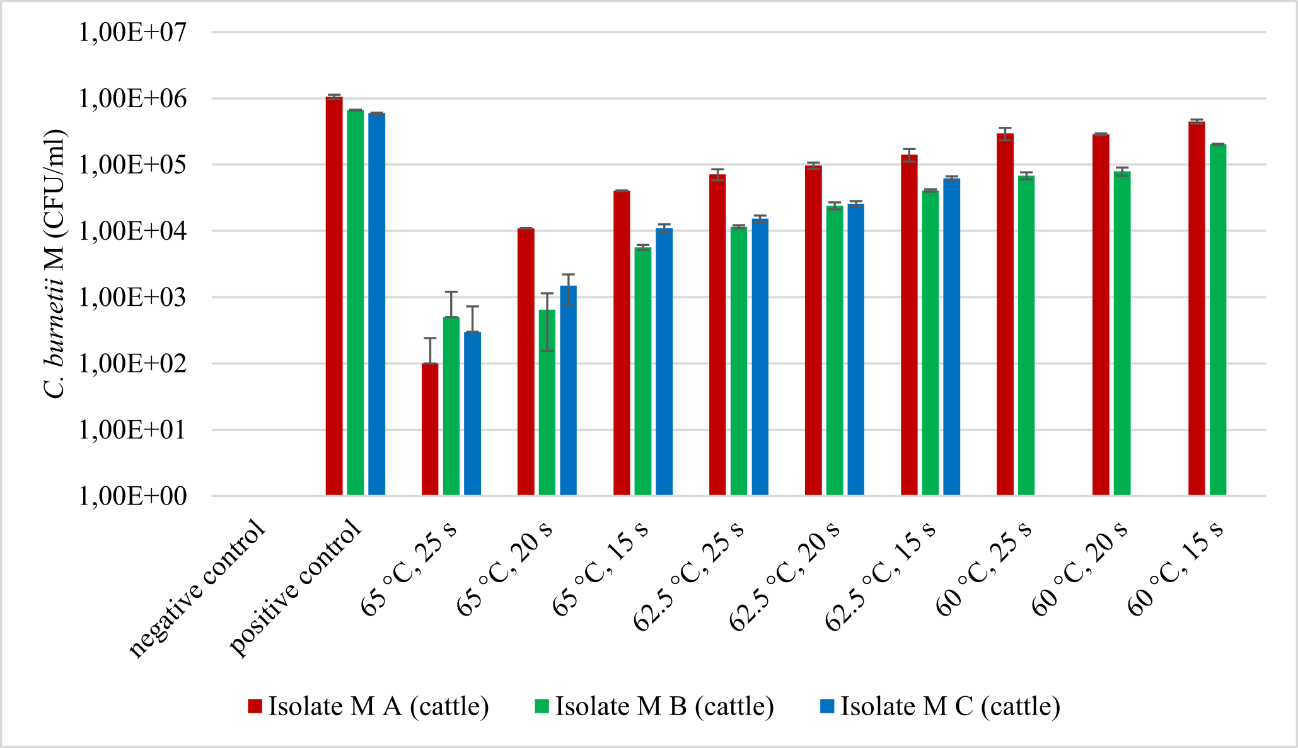


**Fig. VII: Viability testing of *C. burnetii* isolate M using CFU assay, after heat treatment between 60 – 65 °C.** Inoculated UHT milk (3.5% milk fat; 1 x 10^7^ GE/ml) was heat treated at holding times of 15 s, 20 s and 25 s in a pilot plant pasteurizer. Non-heated, inoculated samples served as positive control and non-inoculated UHT milk as negative control. Results represent the calculated average and standard deviation originating from four values, respectively. The displayed pasteurization temperatures were summarized to their intended values, without the individual temperature variations of about ±0.4 °C. Due to experimental complications no data could be obtained for isolate M in experiment C at 60 °C.


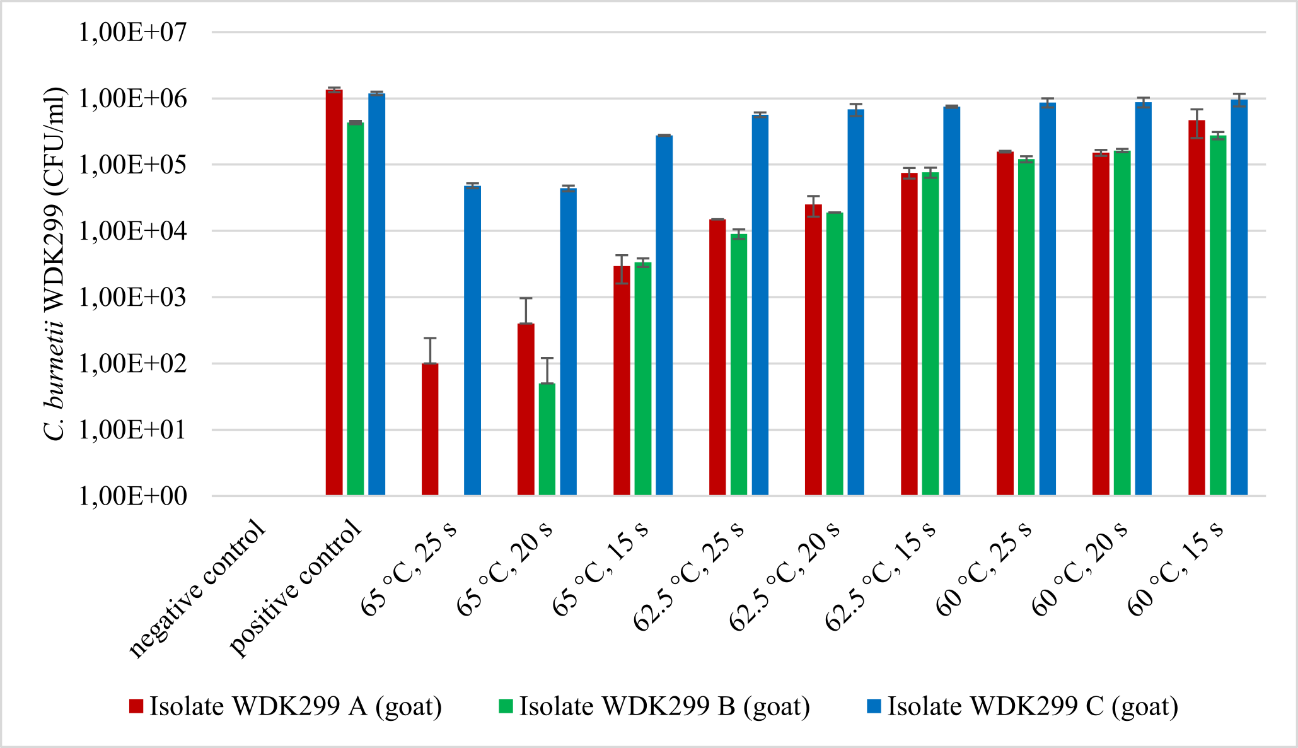


**Fig. VIII: Viability testing of *C. burnetii* isolate WDK299 using CFU assay, after heat treatment between 60 – 65 °C.** Inoculated UHT milk (3.5% milk fat; 1 x 10^7^ GE/ml) was heat treated at holding times of 15 s, 20 s and 25 s in a pilot plant pasteurizer. Non-heated, inoculated samples served as positive control and non-inoculated UHT milk as negative control. Results represent the calculated average and standard deviation originating from four values, respectively. The displayed pasteurization temperatures were summarized to their intended values, without the individual temperature variations of about ±0.1 °C.


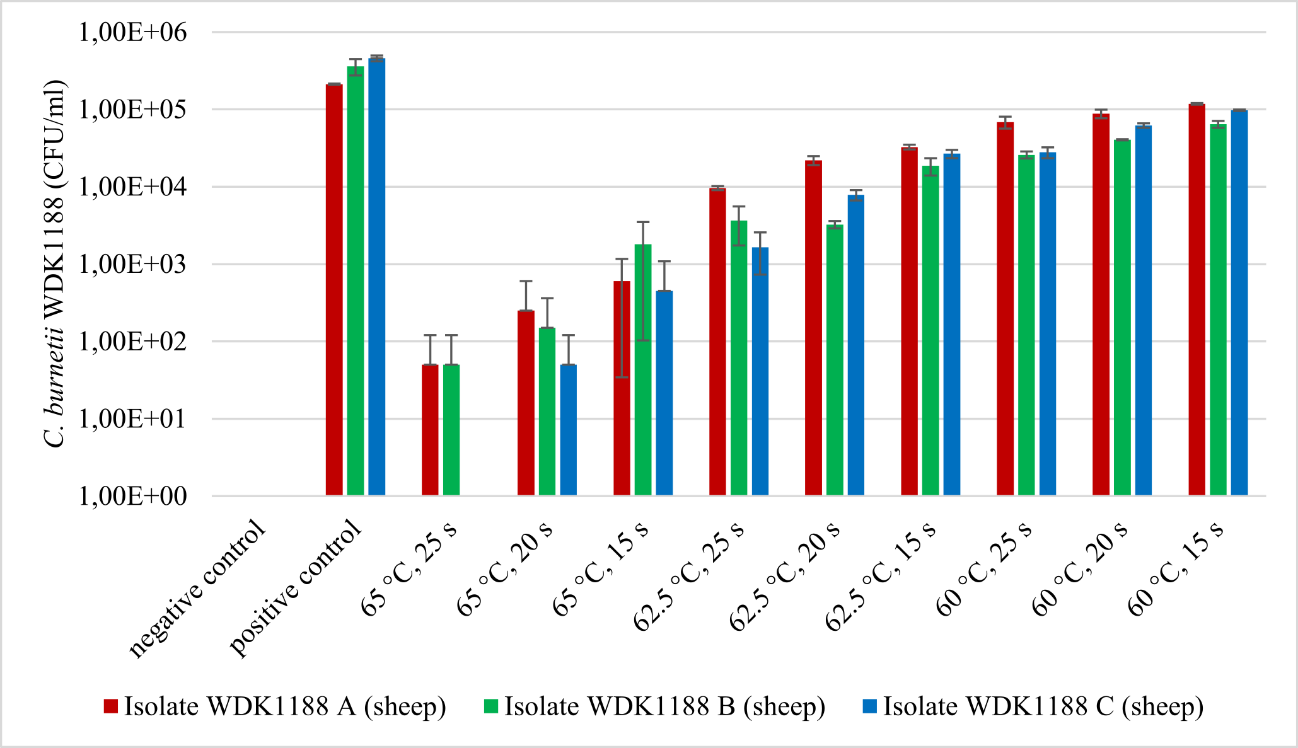


**Fig. IX: Viability testing of *C. burnetii* isolate WDK1188 using CFU assay, after heat treatment between 60 – 65 °C.** Inoculated UHT milk (3.5% milk fat; 1 x 10^7^ GE/ml) was heat treated at holding times of 15 s, 20 s and 25 s in a pilot plant pasteurizer. Non-heated, inoculated samples served as positive control and non-inoculated UHT milk as negative control. Results represent the calculated average and standard deviation originating from four values, respectively. The displayed pasteurization temperatures were summarized to their intended values, without the individual temperature variations of about ±0.2 °C.


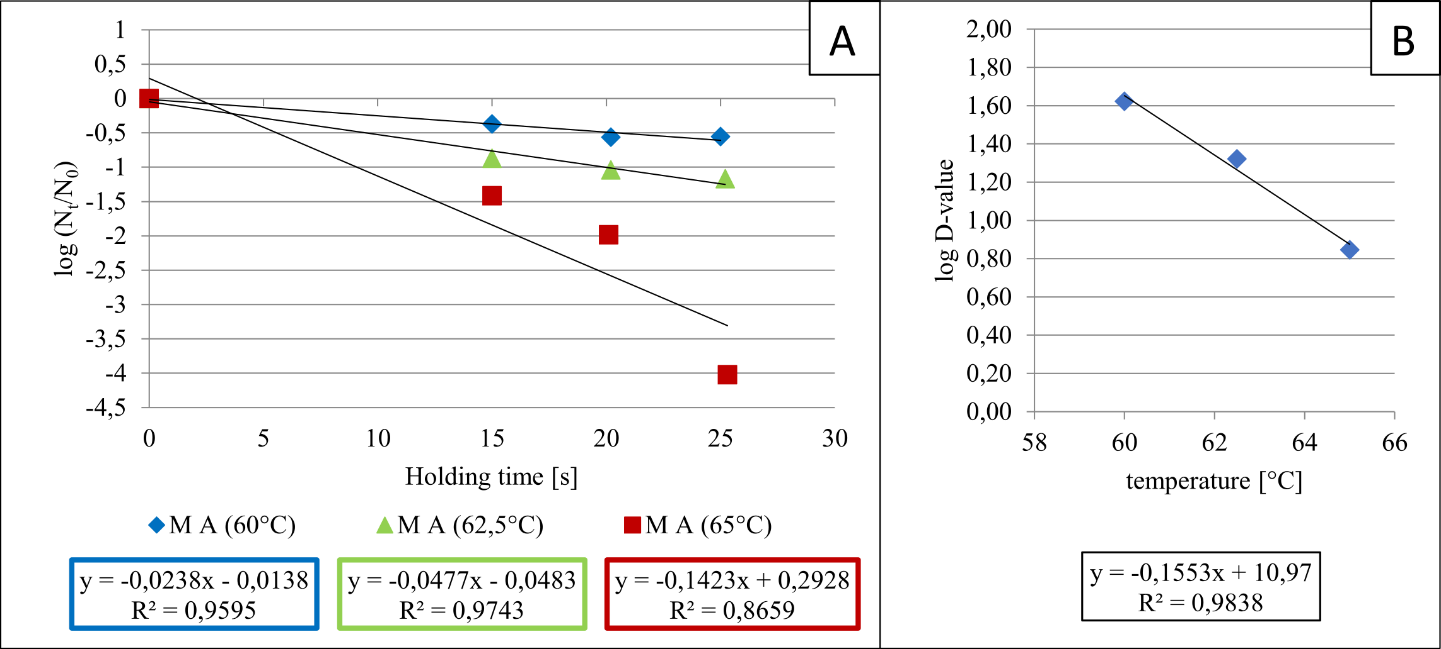


**Fig. X: Portion of the log-transformed *C. burnetii* survivors against the holding time (A) and accordingly portion of the log-transformed D-values against temperature (B) for the cattle isolate M in experiment A.** The slope of the linear equations was used for calculation of the associated D- and z-values (see supplement Table III).


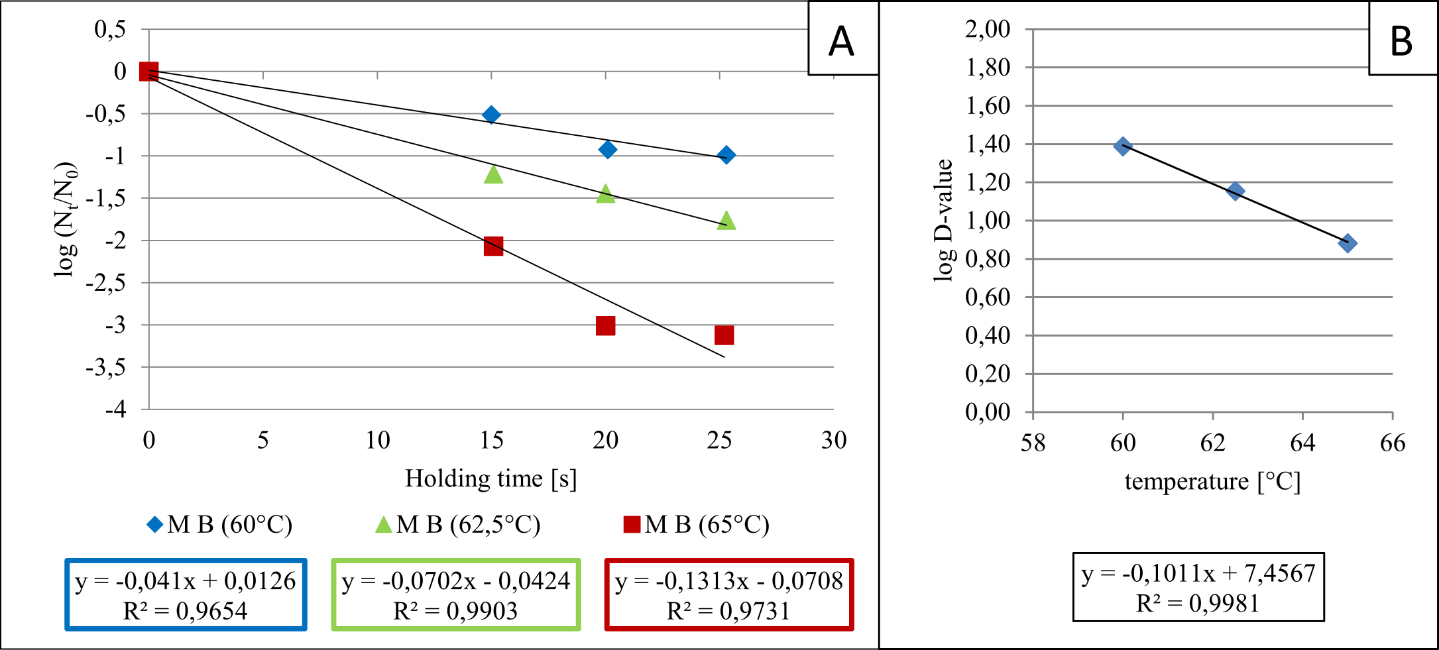


**Fig. XI: Portion of the log-transformed *C. burnetii* survivors against the holding time (A) and accordingly portion of the log-transformed D-values against temperature (B) for the cattle isolate M in experiment B.** The slope of the linear equations was used for calculation of the associated D- and z-values (see supplement Table IV).


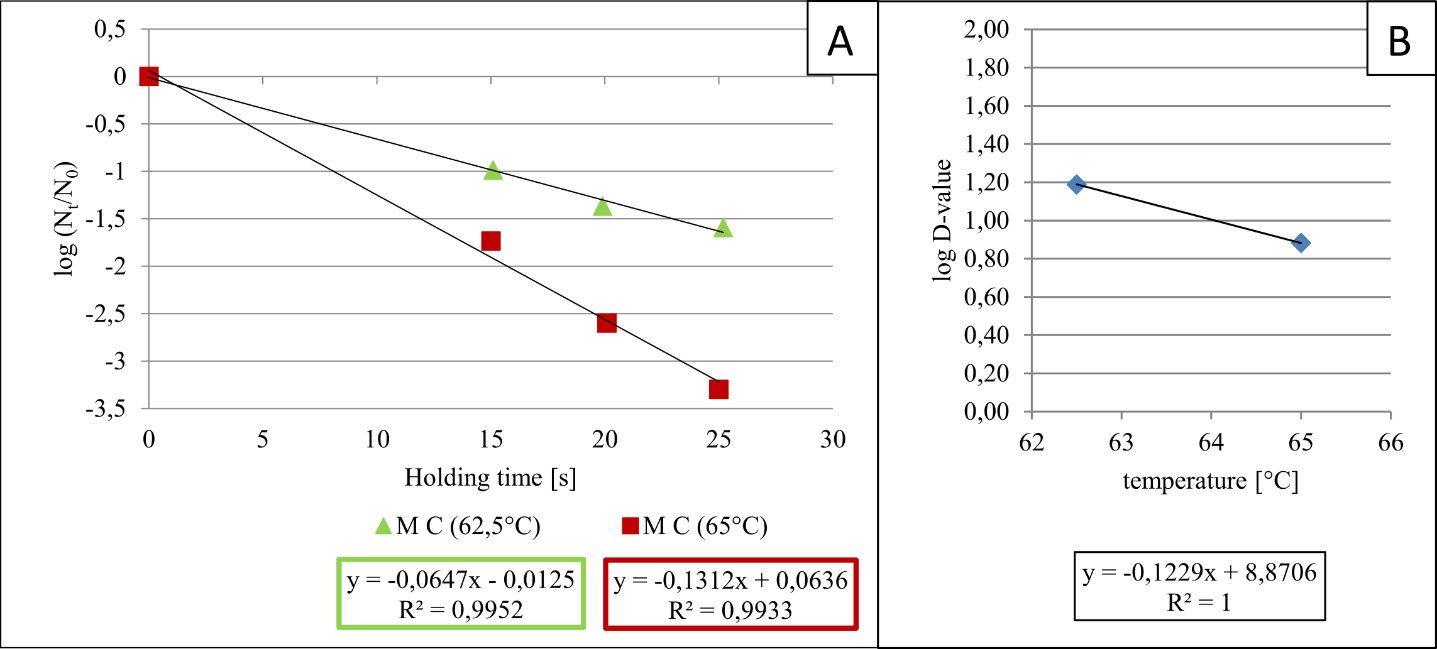


**Fig. XII: Portion of the log-transformed *C. burnetii* survivors against the holding time (A) and accordingly portion of the log-transformed D-values against temperature (B) for the cattle isolate M in experiment C.** The slope of the linear equations was used for calculation of the associated D- and z-values (see supplement Table V).


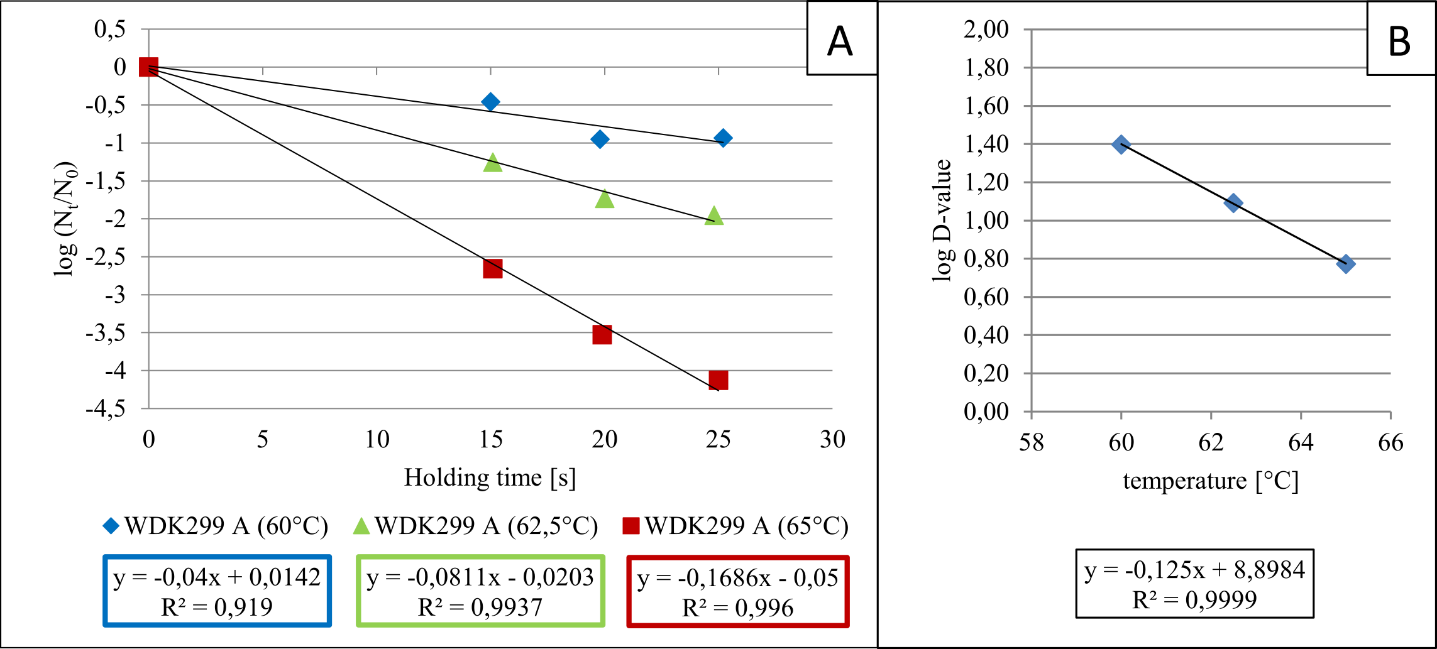


**Fig. XIII: Portion of the log-transformed *C. burnetii* survivors against the holding time (A) and accordingly portion of the log-transformed D-values against temperature (B) for the goat isolate WDK299 in experiment A.** The slope of the linear equations was used for calculation of the associated D- and z-values (see supplement Table VI).


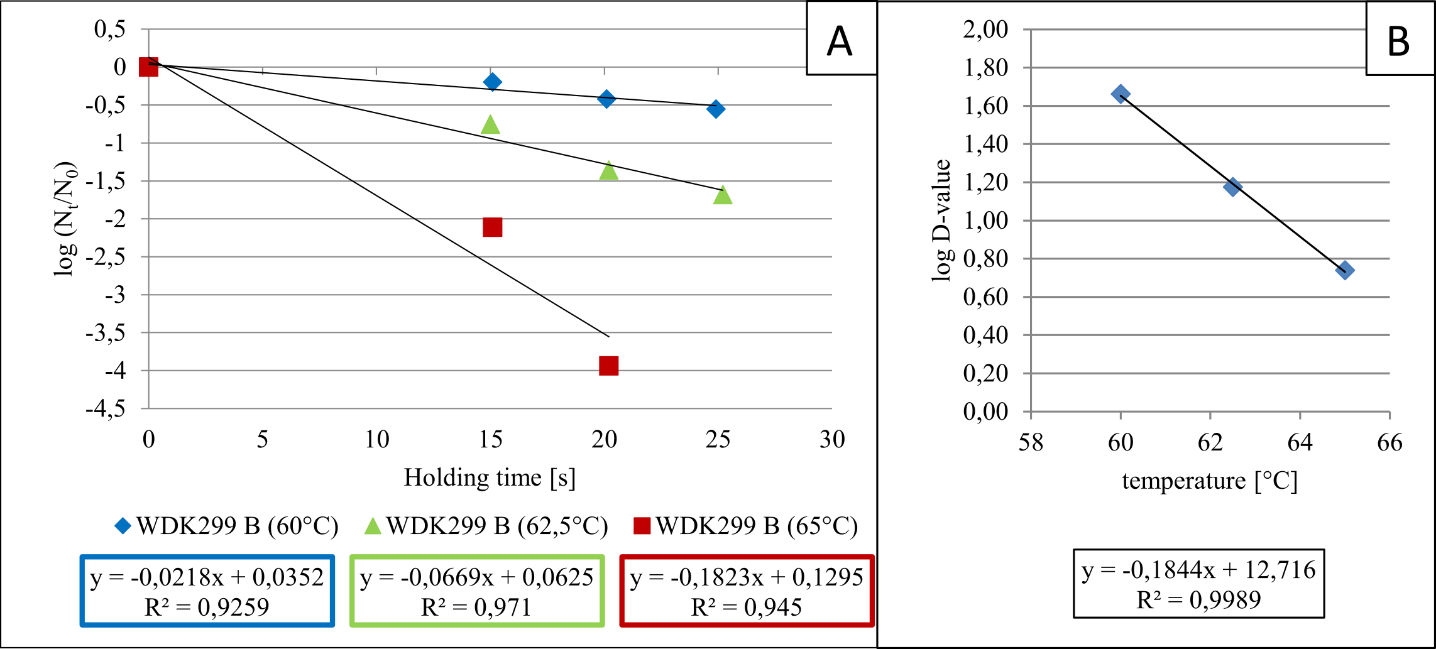


**Fig. XIV: Portion of the log-transformed *C. burnetii* survivors against the holding time (A) and accordingly portion of the log-transformed D-values against temperature (B) for the goat isolate WDK299 in experiment B.** The slope of the linear equations was used for calculation of the associated D- and z-values (see supplement Table VII).


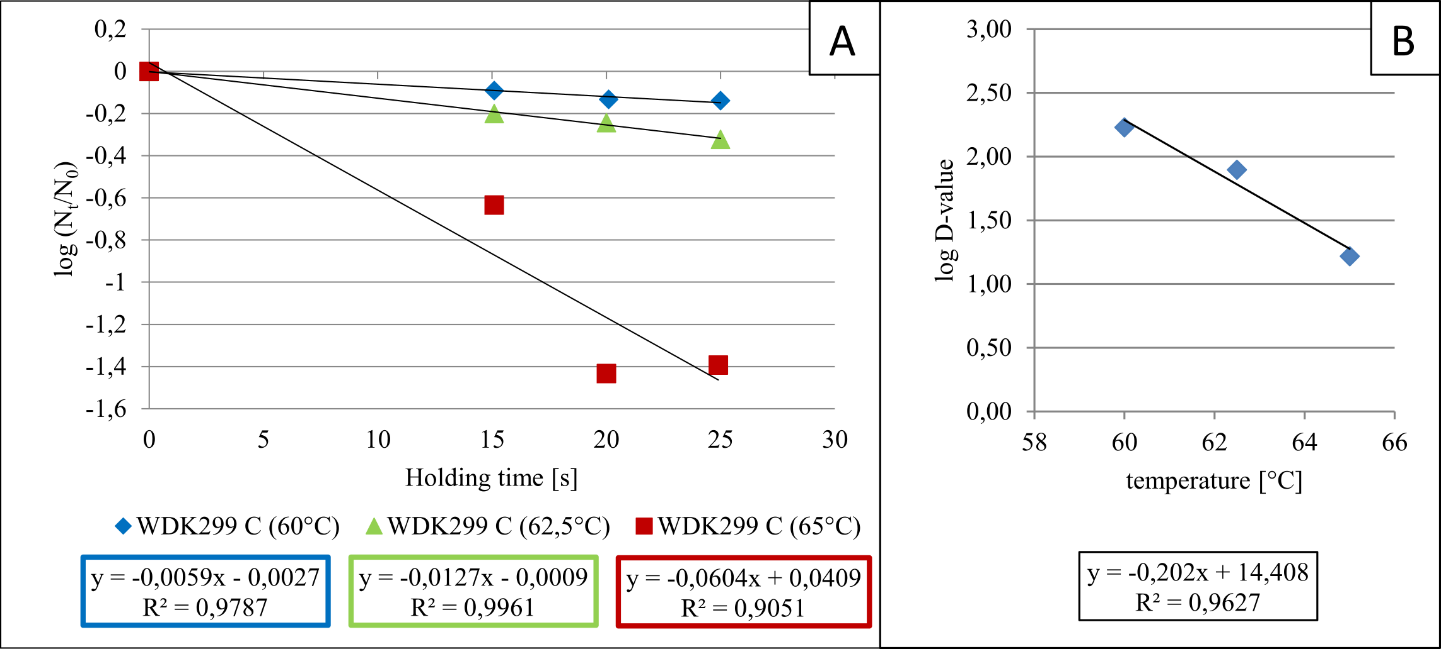


**Fig. XV: Portion of the log-transformed *C. burnetii* survivors against the holding time (A) and accordingly portion of the log-transformed D-values against temperature (B) for the goat isolate WDK299 in experiment C.** The slope of the linear equations was used for calculation of the associated D- and z-values (see supplement Table VIII).


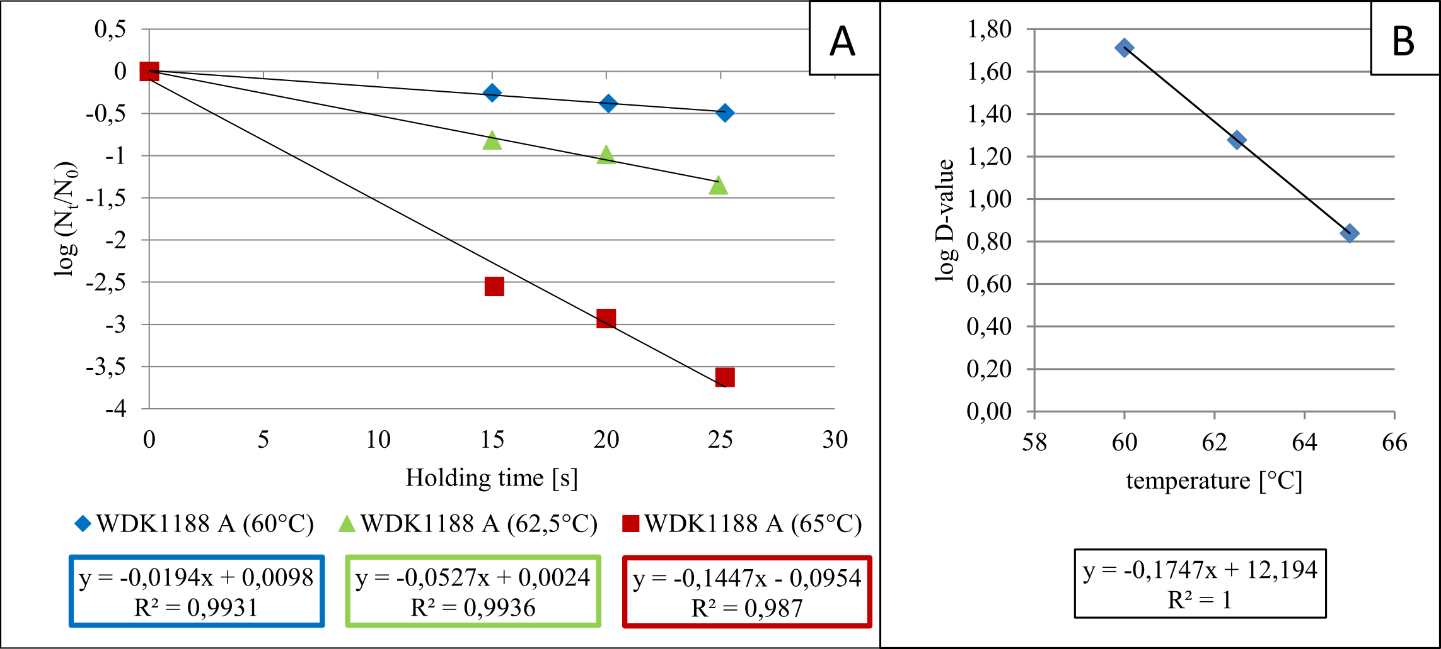


**Fig. XVI: Portion of the log-transformed *C. burnetii* survivors against the holding time (A) and accordingly portion of the log-transformed D-values against temperature (B) for the sheep isolate WDK1188 in experiment A.** The slope of the linear equations was used for calculation of the associated D- and z-values (see supplement Table IX).


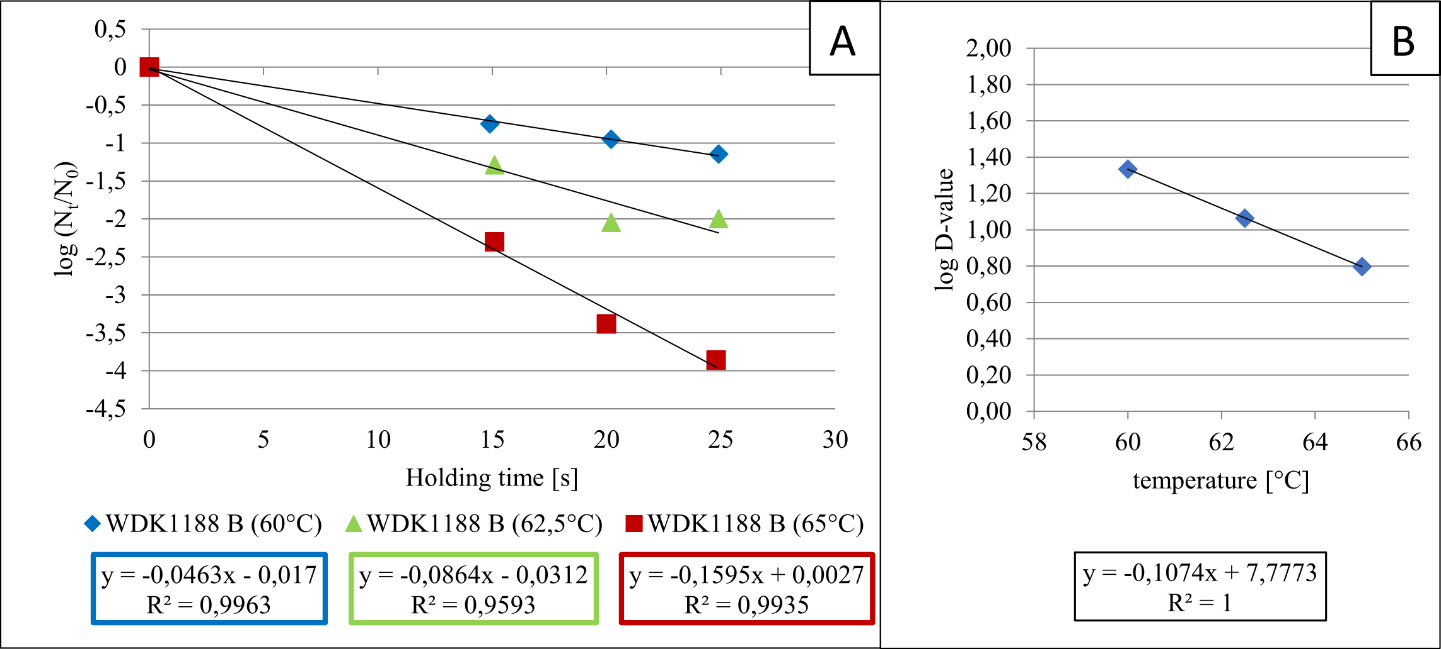


**Fig. XVII: Portion of the log-transformed *C. burnetii* survivors against the holding time (A) and accordingly portion of the log-transformed D-values against temperature (B) for the sheep isolate WDK1188 in experiment B.** The slope of the linear equations was used for calculation of the associated D- and z-values (see supplement Table X).


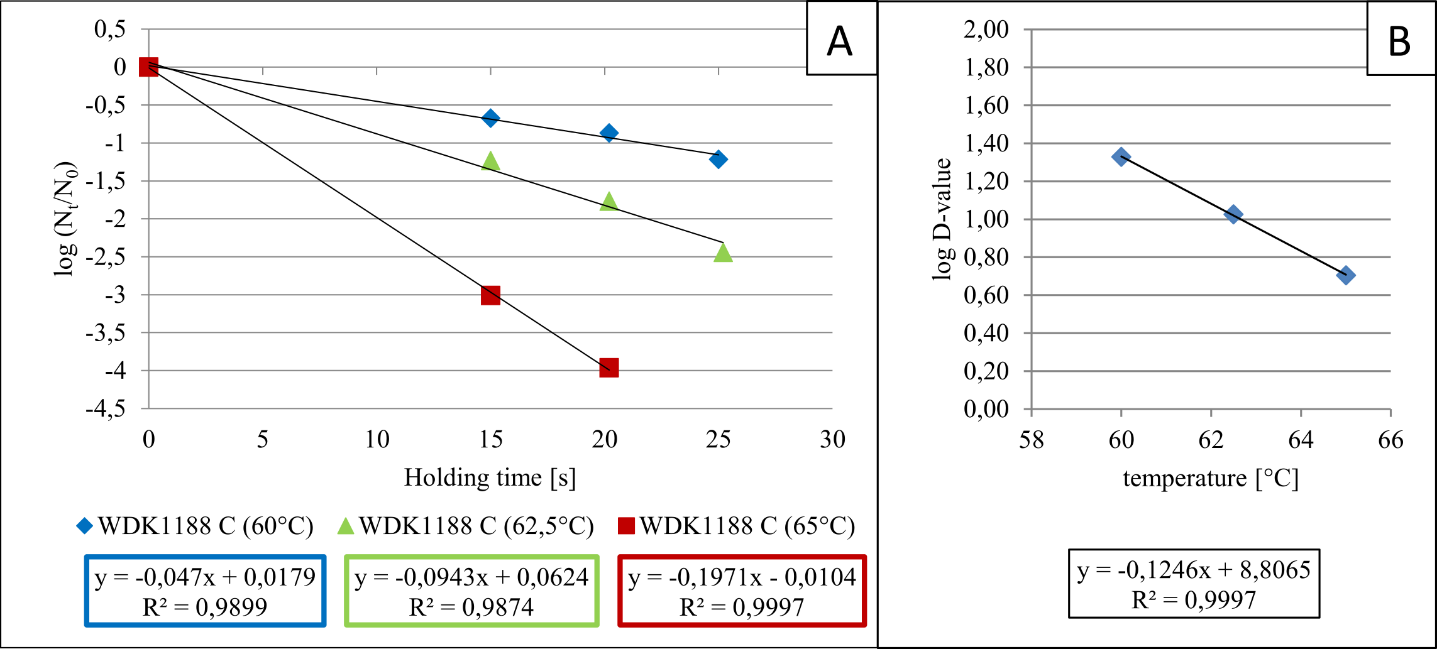


**Fig. XVIII: Portion of the log-transformed *C. burnetii* survivors against the holding time (A) and accordingly portion of the log-transformed D-values against temperature (B) for the sheep isolate WDK1188 in experiment C.** The slope of the linear equations was used for calculation of the associated D- and z-values (see supplement Table XI).

Tables:

**Table I: Calculated U- and p-values of the compared growth curves of all seven isolates.**

|  | **Mann-Whitney U test**  **(U-value)** | |  | **t-test**  **(*p*-value)** | | | |
| --- | --- | --- | --- | --- | --- | --- | --- |
|  | **Isolate**  **M** | **Isolate Bru180** |  | **Isolate WDK299** | **Isolate WDK2932** | **Isolate WDK1188** | **Isolate**  **S1** |
| **Isolate Bru180** | 12 |  |  |  |  |  |  |
| **Isolate WDK299** | 9 | 9 |  |  |  |  |  |
| **Isolate WDK2932** | 9 | 9 |  | 0.88 |  |  |  |
| **Isolate WDK1188** | 2 | 2 |  | 0.33 | 0.26 |  |  |
| **Isolate S1** | 2 | 3 |  | 0.39 | 0.32 | 0.9 |  |
| **NM RSA 439** | 3 | 3 |  | 0.49 | 0.39 | 0.69 | 0.79 |

Quantified growth curve data, gained from their five sampling time points (t=0, 3, 5, 7, 10; GE/ml; see Fig. 1), were used for calculation. Significance determination was carried out by comparison of the growth curve of each isolate with the remaining ones. U-Values greater or rather equal the critical U-Value of 2 as well as *p*-Values above 0.05 confirm the null hypothesis, which means that there is no significant difference in terms of the tested isolates.

**Table II: Growth comparison of the field isolates in cell culture and ACCM-2 by means of their daily log increase.**

| **Isolate** | **Cell culture [daily log increase]** | **ACCM-2 [daily log increase]** | **Factor^1)^** |
| --- | --- | --- | --- |
| M (cattle) | 0.13 | 0.27 | 2.0 |
| Bru180 (cattle) | 0.08 | 0.23 | 2.8 |
| WDK299 (goat) | 0.17 | 0.5 | 3.0 |
| WDK2932 (goat) | 0.12 | 0.47 | 3.9 |
| WDK1188 (sheep) | 0.05 | 0.38 | 7.3 |
| S1 (sheep) | 0.03 | 0.41 | 13.6 |

^1)^factor of advance of *C. burnetii* isolates growing in ACCM-2 compared to their growth in cell culture

**Table III: Summary of relevant data and results for D- and z-value calculation of the cattle isolate M in experiment A.**

| **sample** | **number of colonies** | **log (N_t_/N_0_)** | **D-value**  **(1/m_a_) [s]^1)^** | **log (D-value)** | **z-value**  **(1/m_b_) [°C]^2)^** |
| --- | --- | --- | --- | --- | --- |
| positive control (N_0_) | 1.05E+06 |  |  |  |  |
| 65 °C, 25.3 s (N_t_) | 1.00E+02 | -4.02 |  |  |  |
| 65 °C, 20.1 s (N_t_) | 1.10E+04 | -1.98 |  |  |  |
| 65.1 °C, 15 s (N_t_) | 4.03E+04 | -1.42 | 7.03 | 0.85 |  |
| 62.6 °C, 25.2 s (N_t_) | 7.16E+04 | -1.17 |  |  |  |
| 62.6 °C, 20.2 s (N_t_) | 9.66E+04 | -1.04 |  |  |  |
| 62.5 °C, 15 s (N_t_) | 1.42E+05 | -0.87 | 20.96 | 1.32 |  |
| 60.1 °C, 25 s (N_t_) | 2.96E+05 | -0.55 |  |  |  |
| 60.1 °C, 20.2 s (N_t_) | 2.87E+05 | -0.57 |  |  |  |
| 60 °C, 15 s (N_t_) | 4.48E+05 | -0.37 | 42.02 | 1.62 | 6.4 |

The number of colonies represents the calculated average from four samples, respectively.

^1)^m_a_: slope of the associated linear equation in Fig X A

^2)^m_b_: slope of the associated linear equation in Fig. X B

**Table IV: Summary of relevant data and results for D- and z-value calculation of the cattle isolate M in experiment B.**

| **sample** | **number of colonies** | **log (N_t_/N_0_)** | **D-value**  **(1/m_a_) [s]^1)^** | **log (D-value)** | **z-value**  **(1/m_b_) [°C]^2)^** |
| --- | --- | --- | --- | --- | --- |
| positive control (N_0_) | 6.64E+05 |  |  |  |  |
| 65 °C, 25.2 s (N_t_) | 5.00E+02 | -3.12 |  |  |  |
| 65 °C, 20 s (N_t_) | 6.50E+02 | -3.01 |  |  |  |
| 65 °C, 15.1 s (N_t_) | 5.65E+03 | -2.07 | 7.62 | 0.88 |  |
| 62.5 °C, 25.3 s (N_t_) | 1.16E+04 | -1.76 |  |  |  |
| 62.5 °C, 20 s (N_t_) | 2.41E+04 | -1.44 |  |  |  |
| 62.5 °C, 15.1 s (N_t_) | 4.08E+04 | -1.21 | 14.25 | 1.15 |  |
| 60 °C, 25.3 s (N_t_) | 6.81E+04 | -0.99 |  |  |  |
| 60,1 °C, 20.1 s (N_t_) | 7.92E+04 | -0.92 |  |  |  |
| 60 °C, 15 s (N_t_) | 2.03E+05 | -0.52 | 24.39 | 1.39 | 9.9 |

The number of colonies represents the calculated average from four samples, respectively.

^1)^m_a_: slope of the associated linear equation in Fig XI A

^2)^m_b_: slope of the associated linear equation in Fig. XI B

**Table V: Summary of relevant data and results for D- and z-value calculation of the cattle isolate M in experiment C.**

| **sample** | **number of colonies** | **log (N_t_/N_0_)** | **D-value**  **(1/m_a_) [s]^1)^** | **log (D-value)** | **z-value**  **(1/m_b_) [°C]^2)^** |
| --- | --- | --- | --- | --- | --- |
| positive control (N_0_) | 5.98E+05 |  |  |  |  |
| 65 °C, 25 s (N_t_) | 3.00E+02 | -3.30 |  |  |  |
| 65.2 °C, 20.1 s (N_t_) | 1.50E+03 | -2.60 |  |  |  |
| 64.6 °C, 15 s (N_t_) | 1.11E+04 | -1.73 | 7.62 | 0.88 |  |
| 62.6 °C, 25.2 s (N_t_) | 1.53E+04 | -1.59 |  |  |  |
| 62.5 °C, 19.9 s (N_t_) | 2.57E+04 | -1.37 |  |  |  |
| 62.5 °C, 15.1 s (N_t_) | 6.17E+04 | -0.99 | 15.46 | 1.19 | 8.1 |

The number of colonies represents the calculated average from four samples, respectively.

^1)^m_a_: slope of the associated linear equation in Fig XII A

^2)^m_b_: slope of the associated linear equation in Fig. XII B

**Table VI: Summary of relevant data and results for D- and z-value calculation of the goat isolate WDK299 in experiment A.**

| **sample** | **number of colonies** | **log (N_t_/N_0_)** | **D-value**  **(1/m_a_) [s]^1)^** | **log (D-value)** | **z-value**  **(1/m_b_) [°C]^2)^** |
| --- | --- | --- | --- | --- | --- |
| positive control (N_0_) | 1.35E+06 |  |  |  |  |
| 64.9 °C, 25 s (N_t_) | 1.00E+02 | -4.13 |  |  |  |
| 65 °C, 19.9 s (N_t_) | 4.00E+02 | -3.53 |  |  |  |
| 65 °C, 15.1 s (N_t_) | 2.95E+03 | -2.66 | 5.93 | 0.77 |  |
| 62.5 °C, 24.8 s (N_t_) | 1.50E+04 | -1.95 |  |  |  |
| 62.6 °C, 20 s (N_t_) | 2.50E+04 | -1.73 |  |  |  |
| 62.5 °C, 15.1 s (N_t_) | 7.53E+04 | -1.25 | 12.33 | 1.09 |  |
| 60 °C, 25.2 s (N_t_) | 1.57E+05 | -0.93 |  |  |  |
| 60 °C, 19.8 s (N_t_) | 1.51E+05 | -0.95 |  |  |  |
| 60.1 °C, 15 s (N_t_) | 4.68E+05 | -0.46 | 25 | 1.4 | 8 |

The number of colonies represents the calculated average from four samples, respectively.

^1)^m_a_: slope of the associated linear equation in Fig XIII A

^2)^m_b_: slope of the associated linear equation in Fig. XIII B

**Table VII: Summary of relevant data and results for D- and z-value calculation of the goat isolate WDK299 in experiment B.**

| **sample** | **number of colonies** | **log (N_t_/N_0_)** | **D-value**  **(1/m_a_) [s]^1)^** | **log (D-value)** | **z-value**  **(1/m_b_) [°C]^2)^** |
| --- | --- | --- | --- | --- | --- |
| positive control (N_0_) | 4.33E+05 |  |  |  |  |
| 65 °C, 24.7 s (N_t_) | 0.00E+00 | /^3)^ |  |  |  |
| 65 °C, 20.2 s (N_t_) | 5.00E+01 | -3.94 |  |  |  |
| 65 °C, 15.1 s (N_t_) | 3.35E+03 | -2.11 | 5.49 | 0.74 |  |
| 62.5 °C, 25.2 s (N_t_) | 9.05E+03 | -1.68 |  |  |  |
| 62.5 °C, 20.2 s (N_t_) | 1.90E+04 | -1.36 |  |  |  |
| 62.5 °C, 15 s (N_t_) | 7.66E+04 | -0.75 | 14.95 | 1.17 |  |
| 60 °C, 24.9 s (N_t_) | 1.21E+05 | -0.55 |  |  |  |
| 60 °C, 20.1 s (N_t_) | 1.64E+05 | -0.42 |  |  |  |
| 59.9 °C, 15.1 s (N_t_) | 2.76E+05 | -0.20 | 45.87 | 1.66 | 5.4 |

The number of colonies represents the calculated average from four samples, respectively.

^1)^m_a_: slope of the associated linear equation in Fig XIV A

^2)^m_b_: slope of the associated linear equation in Fig. XIV B

^3)^no replicative *Coxiellas* detected

**Table VIII: Summary of relevant data and results for D- and z-value calculation of the goat isolate WDK299 in experiment C.**

| **sample** | **number of colonies** | **log (N_t_/N_0_)** | **D-value**  **(1/m_a_) [s]^1)^** | **log (D-value)** | **z-value**  **(1/m_b_) [°C]^2)^** |
| --- | --- | --- | --- | --- | --- |
| positive control (N_0_) | 1.19E+06 |  |  |  |  |
| 65 °C, 24.9 s (N_t_) | 4.81E+04 | -1.39 |  |  |  |
| 65 °C, 20 s (N_t_) | 4.39E+04 | -1.43 |  |  |  |
| 65 °C, 15.1 s (N_t_) | 2.76E+05 | -0.63 | 16.56 | 1.22 |  |
| 62.5 °C, 25 s (N_t_) | 5.67E+05 | -0.32 |  |  |  |
| 62.5 °C, 20 s (N_t_) | 6.81E+05 | -0.24 |  |  |  |
| 62.5 °C, 15.1 s (N_t_) | 7.50E+05 | -0.20 | 78.74 | 1.9 |  |
| 60 °C, 25 s (N_t_) | 8.65E+05 | -0.14 |  |  |  |
| 60 °C, 20.1 s (N_t_) | 8.77E+05 | -0.13 |  |  |  |
| 59.9 °C, 15.1 s (N_t_) | 9.63E+05 | -0.09 | 169.49 | 2.23 | 4.9 |

The number of colonies represents the calculated average from four samples, respectively.

^1)^m_a_: slope of the associated linear equation in Fig XV A

^2)^m_b_: slope of the associated linear equation in Fig. XV B

**Table IX: Summary of relevant data and results for D- and z-value calculation of the sheep isolate WDK1188 in experiment A.**

| **sample** | **number of colonies** | **log (N_t_/N_0_)** | **D-value**  **(1/m_a_) [s]^1)^** | **log (D-value)** | **z-value**  **(1/m_b_) [°C]^2)^** |
| --- | --- | --- | --- | --- | --- |
| positive control (N_0_) | 2.13E+05 |  |  |  |  |
| 65 °C, 25.2 s (N_t_) | 5.00E+01 | -3.63 |  |  |  |
| 64.9 °C, 20 s (N_t_) | 2.50E+02 | -2.93 |  |  |  |
| 65 °C, 15.1 s (N_t_) | 6.00E+02 | -2.55 | 6.91 | 0.84 |  |
| 62.5 °C, 24.9 s (N_t_) | 9.60E+03 | -1.35 |  |  |  |
| 62.6 °C, 20 s (N_t_) | 2.20E+04 | -0.99 |  |  |  |
| 62.6 °C, 15 s (N_t_) | 3.27E+04 | -0.81 | 18.97 | 1.28 |  |
| 60 °C, 25.2 s (N_t_) | 6.85E+04 | -0.49 |  |  |  |
| 60 °C, 20.1 s (N_t_) | 8.80E+04 | -0.38 |  |  |  |
| 60 °C, 15 s (N_t_) | 1.18E+05 | -0.26 | 51.55 | 1.71 | 5.7 |

The number of colonies represents the calculated average from four samples, respectively.

^1)^m_a_: slope of the associated linear equation in Fig XVI A

^2)^m_b_: slope of the associated linear equation in Fig. XVI B

**Table X: Summary of relevant data and results for D- and z-value calculation of the sheep isolate WDK1188 in experiment B.**

| **sample** | **number of colonies** | **log (N_t_/N_0_)** | **D-value**  **(1/m_a_) [s]^1)^** | **log (D-value)** | **z-value**  **(1/m_b_) [°C]^2)^** |
| --- | --- | --- | --- | --- | --- |
| positive control (N_0_) | 3.61E+05 |  |  |  |  |
| 64.8 °C, 24.8 s (N_t_) | 5.00E+01 | -3.86 |  |  |  |
| 65 °C, 20 s (N_t_) | 1.50E+02 | -3.38 |  |  |  |
| 64.8 °C, 15.1 s (N_t_) | 1.80E+03 | -2.30 | 6.27 | 0.8 |  |
| 62.6 °C, 24.9 s (N_t_) | 3.65E+03 | -2.00 |  |  |  |
| 62.6 °C, 20.2 s (N_t_) | 3.25E+03 | -2.05 |  |  |  |
| 62.4 °C, 15.1 s (N_t_) | 1.87E+04 | -1.29 | 11.57 | 1.06 |  |
| 60.1 °C, 24.9 s (N_t_) | 2.59E+04 | -1.14 |  |  |  |
| 60.1 °C, 20.2 s (N_t_) | 4.05E+04 | -0.95 |  |  |  |
| 60.1 °C, 14.9 s (N_t_) | 6.43E+04 | -0.75 | 21.59 | 1.33 | 9.3 |

The number of colonies represents the calculated average from four samples, respectively.

^1)^m_a_: slope of the associated linear equation in Fig XVII A

^2)^m_b_: slope of the associated linear equation in Fig. XVII B

**Table XI: Summary of relevant data and results for D- and z-value calculation of the sheep isolate WDK1188 in experiment C.**

| **sample** | **number of colonies** | **log (N_t_/N_0_)** | **D-value**  **(1/m_a_) [s]^1)^** | **log (D-value)** | **z-value**  **(1/m_b_) [°C]^2)^** |
| --- | --- | --- | --- | --- | --- |
| positive control (N_0_) | 4.58E+05 |  |  |  |  |
| 65 °C, 24.8 s (N_t_) | 0.00E+00 | /^3)^ |  |  |  |
| 65.1 °C, 20.2 s (N_t_) | 5.00E+01 | -3.96 |  |  |  |
| 64.9 °C, 15 s (N_t_) | 4.50E+02 | -3.01 | 5.07 | 0.71 |  |
| 62.6 °C, 25.2 s (N_t_) | 1.65E+03 | -2.44 |  |  |  |
| 62.6 °C, 20.2 s (N_t_) | 7.85E+03 | -1.77 |  |  |  |
| 62.5 °C, 15 s (N_t_) | 2.67E+04 | -1.23 | 10.6 | 1.03 |  |
| 60.1 °C, 25 s (N_t_) | 2.79E+04 | -1.22 |  |  |  |
| 60.1 °C, 20.2 s (N_t_) | 6.19E+04 | -0.87 |  |  |  |
| 60 °C, 15 s (N_t_) | 9.75E+04 | -0.67 | 21.28 | 1.33 | 8 |

The number of colonies represents the calculated average from four samples, respectively.

^1)^m_a_: slope of the associated linear equation in Fig XVIII A

^2)^m_b_: slope of the associated linear equation in Fig. XVIII B

^3)^no replicative *Coxiellas* detected
